# BAG3 promotes tumour cell proliferation by regulating EGFR signal transduction pathways in triple negative breast cancer

**DOI:** 10.18632/oncotarget.24590

**Published:** 2018-02-28

**Authors:** Sarah Shields, Emer Conroy, Tony O’Grady, Alo McGoldrick, Kate Connor, Mark P. Ward, Zivile Useckaite, Eugene Dempsey, Rebecca Reilly, Yue Fan, Anthony Chubb, David Gomez Matallanas, Elaine W. Kay, Darran O’Connor, Amanda McCann, William M. Gallagher, Judith A. Coppinger

**Affiliations:** ^1^ UCD School of Biomolecular and Biomedical Science, University College Dublin, Dublin 4, Ireland; ^2^ UCD School of Medicine, University College Dublin, Dublin 4, Ireland; ^3^ Systems Biology Ireland, University College, Dublin 4, Ireland; ^4^ Royal College of Surgeons in Ireland, Dublin 2, Ireland; ^5^ Royal College of Surgeons in Ireland, Beaumont Hospital, Dublin 9, Ireland; ^6^ School of Biology and Environmental Science, University College Dublin, Dublin 4, Ireland

**Keywords:** TNBC, BAG3, EGFR, signalling, proliferation

## Abstract

Triple-negative breast cancer (TNBC), is a heterogeneous disease characterised by absence of expression of the estrogen receptor (ER), progesterone receptor (PR) and lack of amplification of human epidermal growth factor receptor 2 (HER2). TNBC patients can exhibit poor prognosis and high recurrence stages despite early response to chemotherapy treatment. In this study, we identified a pro-survival signalling protein BCL2- associated athanogene 3 (BAG3) to be highly expressed in a subset of TNBC cell lines and tumour tissues. High mRNA expression of BAG3 in TNBC patient cohorts significantly associated with a lower recurrence free survival. The epidermal growth factor receptor (EGFR) is amplified in TNBC and EGFR signalling dynamics impinge on cancer cell survival and disease recurrence. We found a correlation between BAG3 and EGFR expression in TNBC cell lines and determined that BAG3 can regulate tumour cell proliferation, migration and invasion in EGFR expressing TNBC cells lines. We identified an interaction between BAG3 and components of the EGFR signalling networks using mass spectrometry. Furthermore, BAG3 contributed to regulation of proliferation in TNBC cell lines by reducing the activation of components of the PI3K/AKT and FAK/Src signalling subnetworks. Finally, we found that combined targeting of BAG3 and EGFR was more effective than inhibition of EGFR with Cetuximab alone in TNBC cell lines. This study demonstrates a role for BAG3 in regulation of distinct EGFR modules and highlights the potential of BAG3 as a therapeutic target in TNBC.

## INTRODUCTION

Triple-negative breast cancer (TNBC), is a heterogeneous disease characterised by negative expression of the estrogen receptor (ER), progesterone receptor (PR) and lack of overexpression of the human epidermal growth factor receptor 2 (HER2) [1]. TNBC patients can exhibit poor prognosis and high relapse rates at early stages after adjuvant and neoadjuvant chemotherapy treatment [2, 3]. New targeted therapies are currently being explored. The epidermal growth factor receptor (EGFR) is known to be over expressed in >50% of TNBC patients [4]. However, inhibiting EGFR alone in TNBC has had limited efficacy [5], possibly due to the presence of additional mutations in downstream EGFR signaling nodes such as KRAS and PTEN. Encouragingly, combination therapies targeting multiple signalling nodes have proved more successful than single target therapies [6, 7].

Chaperones expressed at high levels in mammary carcinoma, can play key roles in tumour progression and have become important targets for therapy [8]. The BAG-family of co-chaperones can regulate cellular behaviours including cell migration and differentiation by operating as bridging molecules that recruit molecular chaperones to target proteins that modulate their function [9]. In this manuscript, we investigate a role for the BCL2- Associated Athanogene 3 (BAG3) gene in driving cancer cell proliferation by regulating signalling pathways in TNBC. BAG3 is a member of the BAG family of co-chaperones and the multi modular composition of BAG3 allows a wide range of protein–protein interactions linking BAG3 to several key pathways in the cell [10]. This protein contains the BAG domain that binds to a motif in the ATPase domain of Hsp70 proteins, as well as a WW domain that provides a platform for the assembly of multiprotein networks [11]. Additionally BAG3 contains two Ile-Pro-Val (IPV) motifs that allow binding of BAG3 to the small heat shock proteins and a PXXP motif [10] which is a docking site for SH3 (Src homology 3) domains present in proteins such as phospholipase Cγ (PLCγ) [12], and other important signalling proteins [13].

BAG3 has been shown to be overexpressed in tumour cell lines and solid tumours including small cell lung carcinomas, glioblastomas and pancreatic adenocarcinomas where it’s overexpression is associated with poor survival [14–16]. BAG3 has also been shown to regulate cell proliferation and motility in different cancer cell lines [17, 18] by regulating the migratory cellular phenotype through interaction with SH3 domain-containing proteins involved in focal adhesion formation [19]. Additionally BAG3 has been reported to regulate epithelial-mesenchymal transition (EMT) and metastasis in a variety of cancer models [20–22].

In this study we investigated a role for BAG3 in driving cancer cell proliferation in TNBC models by stabilising EGFR signalling nodes. We identified an interaction between BAG3 and components of the EGFR signalling pathways using mass spectrometry based proteomics. Furthermore, we have shown BAG3 contributes to the regulation of proliferation in TNBC cell lines through the AKT and FAK signalling pathways. We determined that targeting of BAG3 using a BAG3-HSP70 protein interaction inhibitor significantly improved therapeutic response in TNBC compared to EGFR inhibition alone.

## RESULTS

### BAG3 is expressed in TNBC cell lines and patient tissues

**Figure 1:**
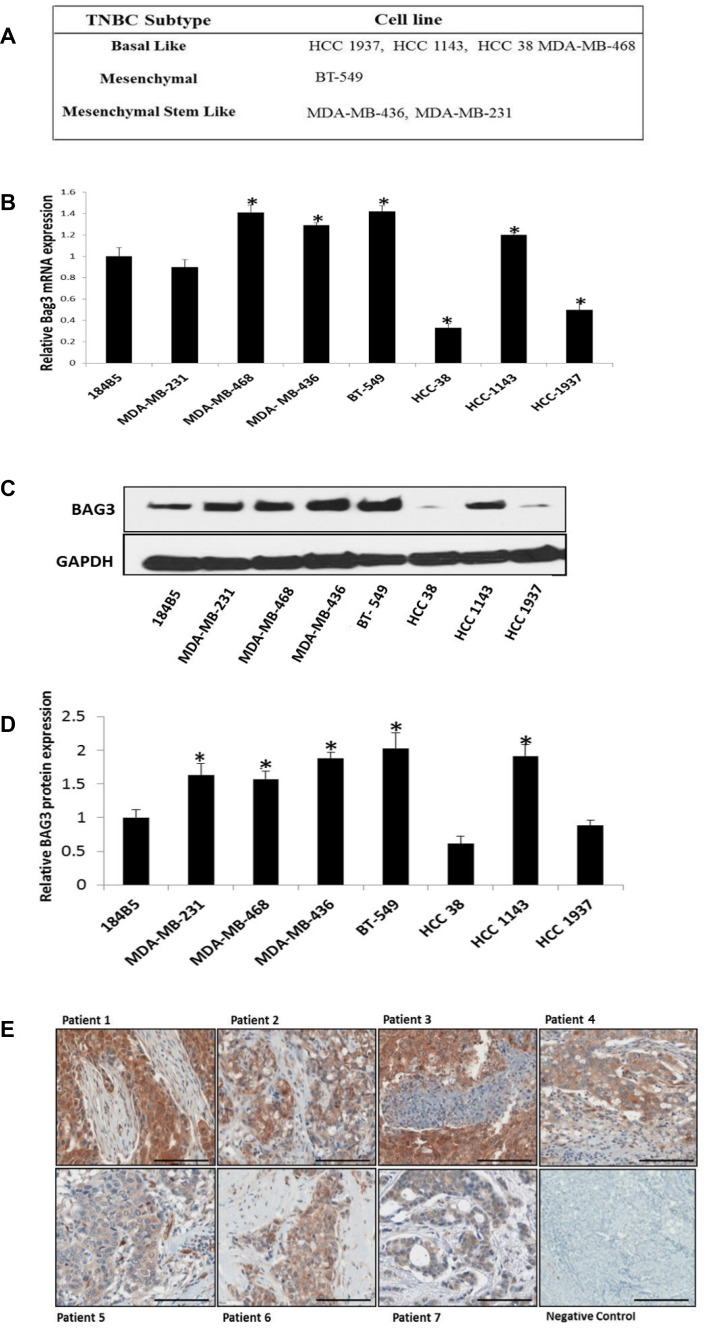
BAG3 is expressed in TNBC cell lines and patient tissues (**A**) 7 heterogeneous TNBC cell lines and their predicted subtypes are listed. (**B**) A quantitative graph of BAG3 mRNA expression in 7 TNBC cells lines and one normal breast epithelial cell lines 184B5. Experiments were performed in triplicate and normalised against GAPDH. The histograms represent the average mRNA expression of BAG3 (± SD). An asterisk represents *p* < 0.05. (**C**) The protein expression levels of BAG3 in 7 TNBC cell lines and 1 normal breast epithelial cell line 184B5 analyed by immunoblotting. (**D**) A quantitative graph of BAG3 protein expression in TNBC cell lines. The results shown are representative of three independent experiments. The histograms represent the average expression of BAG3 (± SD). (**E**) Representative scanned images of TNBC tumour sections with high (top panel) or low (bottom panel) BAG3 expression as determined by immunohistochemistry. Scale bar is 100 uM. Magnification is 20×.

We determined BAG3 RNA and protein expression in a panel of TNBC cell lines. This panel represented TNBC of both a basal and mesenchymal origin with both ductal and invasive ductal histologies (Figure 1A) (Supplementary Figure 1A). A normal breast epithelial cell line was included as a control (184B5). BAG3 expression was detected at both the RNA (Figure 1B) and protein level (Figure 1C, 1D) in the TNBC cell line panel. There was a significant increase in BAG3 expression at both the RNA and protein level in four TNBC cell lines (MDA-MB-468, MDA-MB-436, BT-549 & HCC1143) relative to the control. These cell lines were invasive with the exception of the MDA-MB-468 cell line. A decrease in BAG3 RNA and protein expression was observed in the basal non-invasive cell lines HCC1937 and HCC38 (Figure 1B, 1D). There was some discordance between the RNA and protein expression levels of BAG3 in the MDA-MB-231 cell line. However, given that post-transcriptional regulation often leads to generally low correlation between mRNA and protein concentrations [23] the results we obtained for BAG3 mRNA and BAG3 protein expression levels in the TNBC cell line panel were positive with six cell lines showing a similar trend. The expression of BAG3 was also examined in a clinical cohort (Cohort 3) of TNBC patients (*n* = 80) with associated clinical outcome data (Figure 1E) (Supplementary Figure 1B). Histopathological review of the sections revealed increased tumour epithelial BAG3 expression was demonstrable in 37/80 cases compared to the surrounding stromal tissue and these were graded as high BAG3 expressing tissues (Score 2, 3). 41 were graded as low BAG3 (Score 0, 1) expressing tissues. Two of the sections analysed had insufficient tumour present to assess BAG3 expression (Figure 1E). In conclusion, we observed high BAG3 expression in a subset (∼50%) of the TNBC cell lines and patient tissues analysed.

### High BAG3 mRNA expression correlates with poorer disease free survival

**Figure 2:**
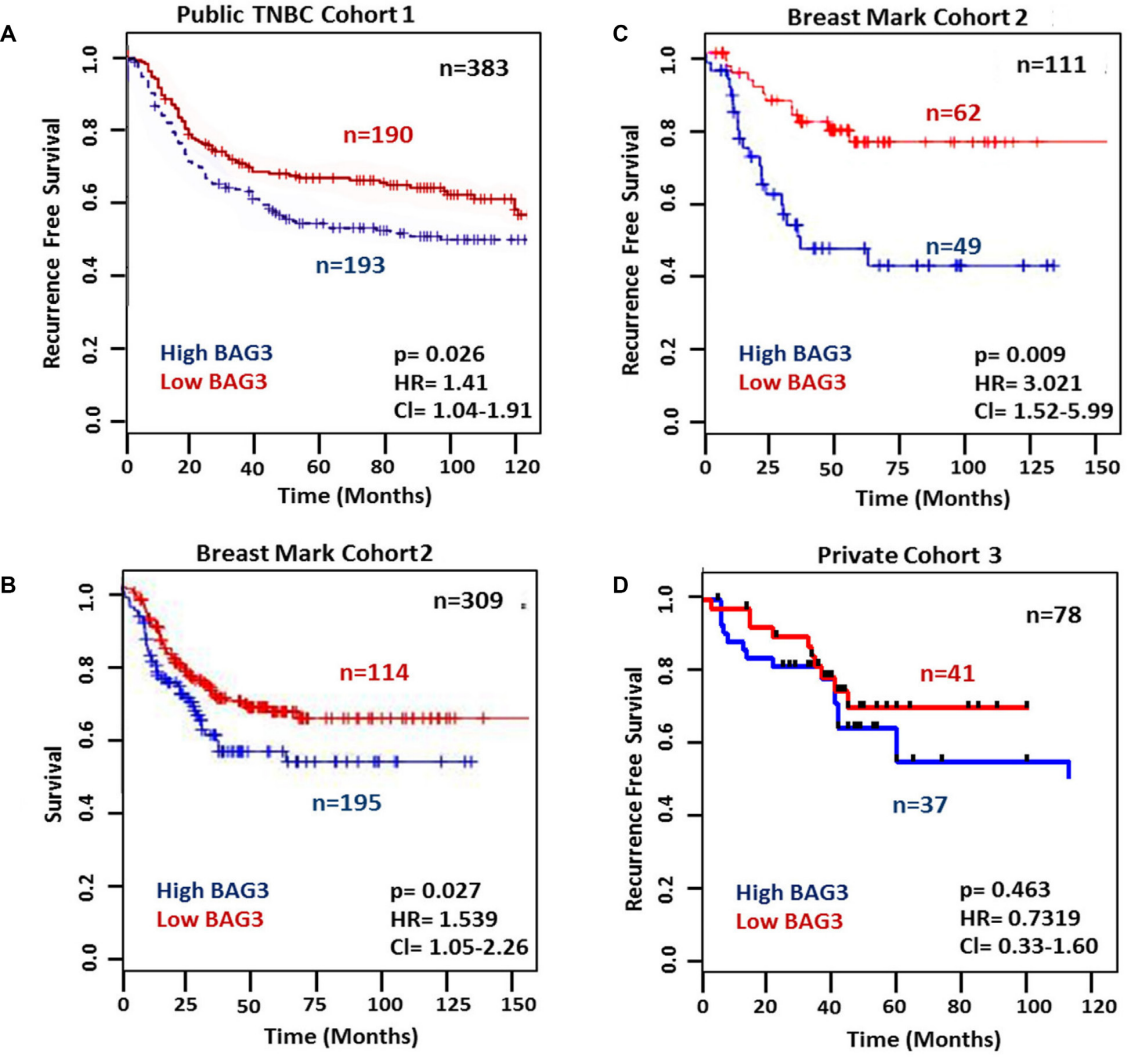
High BAG3 mRNA expression correlates with poorer disease free survival (**A**) Kaplan-Meier survival curves showing the relationship between BAG3 mRNA expression and recurrence free survival (RFS) in a publicly available TNBC dataset censored at 10 years (*n* = 383). (**B**) Kaplan–Meier survival curves showing the relationship between BAG3 mRNA expression and survival using *Breast Mark* filtered cancer datasets (*n* = 309). (**C**) Kaplan–Meier survival curves showing the relationship between BAG3 mRNA expression and RFS using publicly available *Breast Mark* filtered cancer datasets (*n* = 111). (**D**) Kaplan–Meier survival curves showing the relationship between BAG3 protein expression and RFS in a private cohort of 78 TNBC patients (*n* =78). A two-tailed test with *P* value < 0.05 was considered to be significant. Kaplan Meier survival analysis with Log-rank (Mantel-Cox) testing was employed to investigate if the BAG3 gene was significantly associated with RFS. Cox regression analysis was used to calculate hazard ratios in all Cohorts.

In order to determine whether BAG3 expression correlated with disease free survival in clinical datasets we used two different approaches. Firstly we examined publicly available transcriptomic data from a combined cohort of 579 TNBC patients, of which 383 had associated clinical outcome data [24] (Cohort 1). When a median BAG3 mRNA expression cut-off point was used for stratification, 193 patients had high BAG3 mRNA expression which significantly correlated with reduced recurrence free survival (RFS) [*P* =0.026, HR = 1.41, Cl (1.04–1.91)] (Figure 2A). Further survival analysis was carried out using *BreastMark*, an online integrated resource [25] to allow evaluation of genes that are associated with survival outcome in breast cancer. No significant correlation between BAG3 mRNA and RFS was observed in the unstratified cohort, which contains 2,656 breast cancer patient samples (data not shown). However, when custom analysis was performed within this cohort (Cohort 2) for TNBC subset ER/PR/HER2 negative breast cancer patients (*n* = 309), a significant correlation was observed between high BAG3 mRNA expression and reduced overall survival, [*p* = 0.027, HR = 1.539, Cl (1.05–2.26)] (Figure 2B) and reduced recurrence free survival [*p* = 0.009, HR = 3.021, Cl (1.52–5.99)] (Figure 2C). In order to determine if BAG3 expression at the protein level correlated with poorer disease free survival we analysed a third cohort of patients (Cohort 3) (Figures 1E, 2D). Although ∼50% of patients again showed high BAG3 protein expression, no significant observation between BAG3 and recurrence free survival was observed in this patient set [*p* = 0.463, HR = 0.7319, Cl (0.33–1.60)]. Due to the small number of patients with recurrent disease in this clinical cohort (*n* = 25), further validation of BAG3 protein expression in a larger TNBC cohort is warranted. Overall, these initial studies suggest a potential role for BAG3 mRNA as a prognostic marker of disease recurrence in TNBC.

### BAG3 regulates cell proliferation, migration and invasion in TNBC cell lines

**Figure 3:**
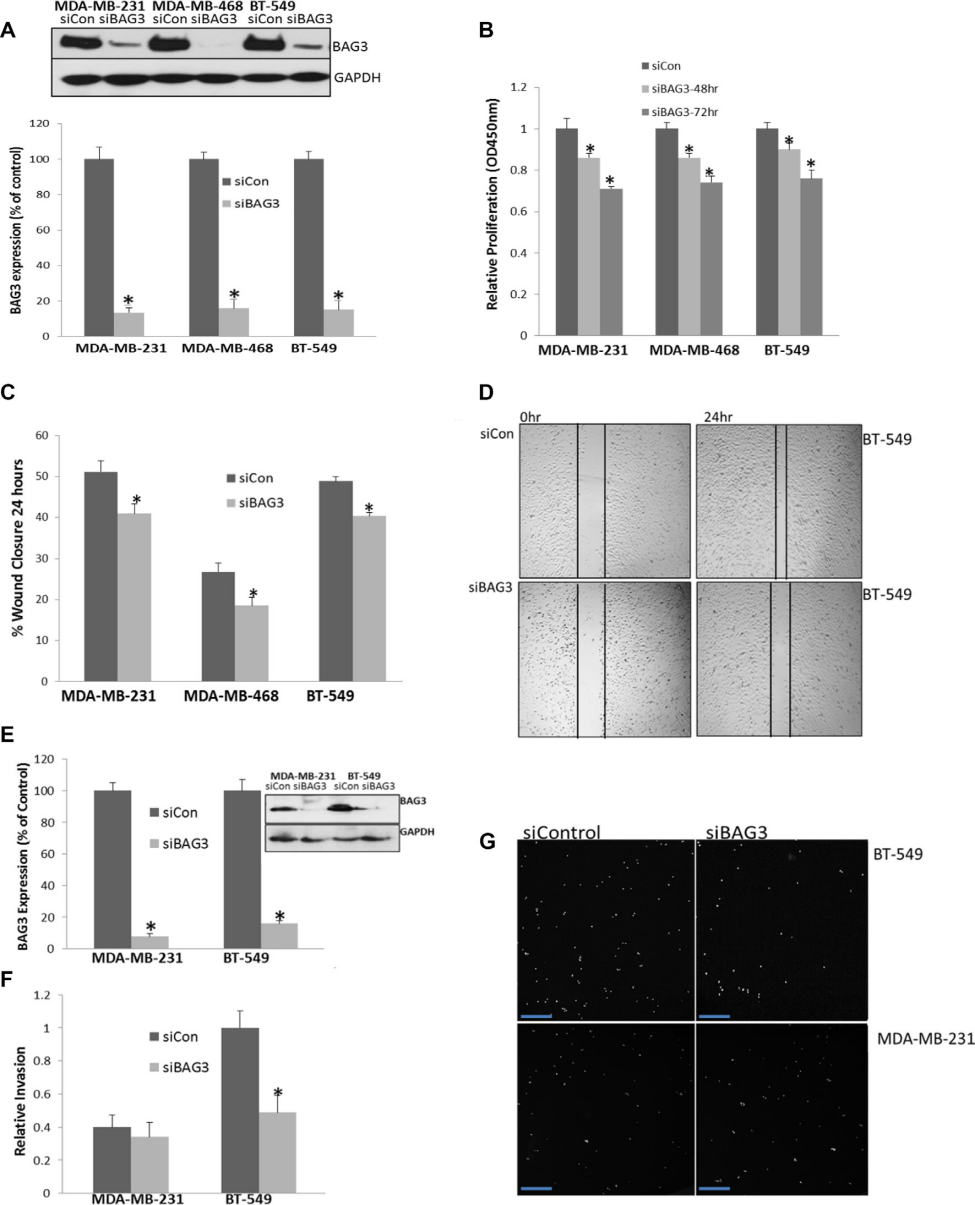
BAG3 regulates cell proliferation, migration and invasion in TNBC cell lines (**A**) MDA-MB-468, MDA-MB-231 and BT-549 cells were treated with siControl and siBAG3 and immunoblotting and densitometry was performed for BAG3 and GAPDH in triplicate. Quantification of BAG3 knockdown efficiency was calculated as % of the control. (**B**) Proliferation was measured after 48 and 72 hrs in MDA-MB-468, MDA-MB-231 and BT-549 cells treated with siBAG3 and siControl. The histograms represent mean ( ± SD) Brdu incorporation relative to the control (*n* = 3). An asterisk represents *p* < 0.05. (**C**) A quantitative graph of percentage wound closure from MDA-MB-468, MDA-MB-231 and BT-549 cells treated with either siBAG3 or siControl. The histograms represent % wound closure (± SD) in triplicate. An asterisk represents *p* < 0.05. (**D**) Scratch wound illustration of BT-549 cells after a 24 hour migration period. (**E**) MDA-MB-231 and BT-549 cells were treated with siControl and siBAG3 and immunoblotting and densitometry was performed for BAG3 and GAPDH in triplicate (**F**) A quantitative graph of relative invasion from MDA-MB-231 and BT-549 cells after 24 hrs with either siBAG3 or siControl. The histograms represent the number of invasive cells detected relative to control (± SD) in triplicate. An asterisk represents *p* < 0.05. (**G**) Random selected fields of invaded cells from MDA-MB-231 and BT-549 cells after treatment with either siBAG3 or siControl. Scale bars are 100 µM.

BAG3 has previously been shown to regulate cell motility and migration in epithelial cancer cell lines [17]. In order to determine if BAG3 could regulate cell tumour proliferation in TNBC, the BAG3 gene was gene silenced in three TNBC cell lines that expressed high levels of BAG3 protein (MDA-MB-231, MDA-MB-468 and BT-549 cells) and the quantification efficiency of reduced BAG3 protein expression measured by densitometry (>80%) (Figure 3A). Cellular proliferation was then measured by the incorporation of BrdU. This was performed in triplicate at both 48 and 72 hours post BAG3 gene silencing. A significant decrease in cell proliferation was observed in all the three cell lines relative to the control at both time points with a more pronounced reduction at 72 hours after siRNA treatment (Figure 3B) (*p* < 0.05). However we observed a significant level of apoptosis at the 72 hour time point (Supplementary Figure 2). As we were interested in studying pathways leading to proliferation not apoptosis in this study we pursued the 48 hour time point for subsequent experiments where minimal cell death was observed. To ensure siRNA specificity, supplementary experiments were performed using additional siRNAs and a mutated sequence (C9-C11) targeting BAG3. There was no significant reduction in proliferation in TNBC cell lines with the C9-C11 form of the siRNA selected (Supplementary Figure 3). In order to control for these experiments further, we overexpressed BAG3 in a cell line expressing low levels of BAG3 (HCC1937) and observed an increase in proliferation upon overexpression of BAG3 (Supplementary Figure 4A). We then analysed cell migration in the same TNBC cell lines treated with siBAG3. Cell migration was assessed with a wound healing scratch assay and cell migratory rates were quantified in triplicate. Supplementary immunofluorescence experiments were performed to validate the presence of migrating cells using this technique (Supplementary Figure 5). A significant decrease in cell motility was observed in the three TNBC cell lines relative to the control (*p* < 0.05) (Figure 3C). A representative image of wound closure after BAG3 silencing in BT-549 cells is displayed (Figure 3D). As an additional control we examined if BAG3 could regulate migration in HCC1937 cell lines. BAG3 was overexpressed in this cell line as it is a low BAG3 expressing cell line and cell motility determined as before. There was a significant increase in cell motility (wound closure) when BAG3 was overexpressed relative to the control (Supplementary Figure 4B). In order to determine if BAG3 may also regulate invasion in these cell lines, a Boyden chamber assay was employed and cells invading through the matrigel quantified by fluorescence. We observed minimal invasion in MDA-MB-468 cells (data not shown). Therefore, further analysis was performed in MDA-MB-231 and BT-549 cell lines. BAG3 was targeted by gene silencing as before and the quantification efficiency of reduced BAG3 protein expression measured by densitometry (>85%) (Figure 3E). A decrease in cell invasion was observed in both cell lines (Figure 3F). Statistical analysis revealed this to be significant in BT-549 cells (*p* < 0.05) (*n* = 3). A representative fluorescent field is shown for all conditions (Figure 3G). Collectively these results indicate that gene silencing of BAG3 results in reduced, proliferation, migration and invasion in TNBC cell subtypes indicating that inhibiting BAG3 could be of therapeutic value in TNBC.

### BAG3 interacts with EGFR and components of the EGFR pathway

**Figure 4:**
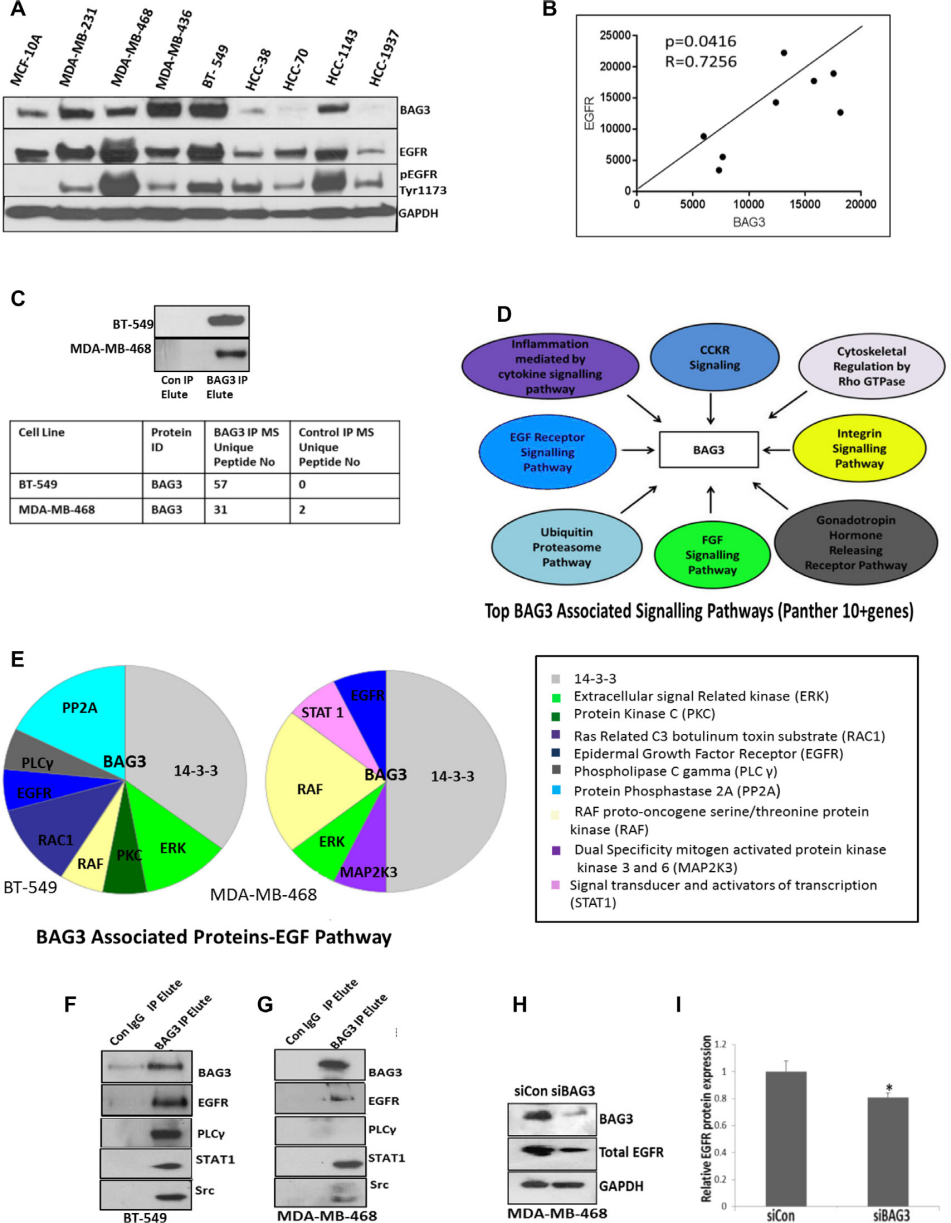
BAG3 interacts with EGFR and components of the EGFR pathway (**A**) Protein expression levels of BAG3, pEGFRTyr1173, EGFR and GAPDH in 8 TNBC cell lines and 1 normal breast epithelial cell line 184B5 analysed by immunoblotting. (**B**) Pearson Correlation Analysis for BAG3 versus EGFR expression in 8 TNBC cell lines is displayed. (**C**) BAG3 was immunoprecipitated (IP) from MDA-MB-468 and BT-549 cells and enrichment of BAG3 by immunoblotting is displayed. The number of BAG3 peptides enriched in the IP identified by mass spectrometry are also displayed (table). (**D)** Proteins identified in the BAG3 Interactome (IP) by mass spectrometry were analysed using the PANTHER Classification System. The top 8 Panther Pathways (as determined by gene enrichment) represented in the BAG3 Interactome are listed (**E**) EGF signalling proteins identified in the BAG3 Interactome of BT-549 and MDA-MB-468 cells are listed (**F**) Immunoblotting was performed for BAG3, EGFR, PLCγ, STAT1 and Src in the BAG3 Interactome isolated from BT-549 cells (**G**) Immunoblotting was performed for BAG3, EGFR, PLCγ, STAT1 and Src in the BAG3 Interactome isolated from MDA-MB-468 cells. (**H**) The protein expression of BAG3, EGFR and GAPDH in MDA-MB-468 cells treated with siBAG3 and siControl. (**I**) A quantitative graph of EGFR protein expression in MDA-MB-468 cells treated with siBAG3 and siControl. The histograms represent the average protein expression of EGFR (± SD) (*n* = 3).

Due to previous work, we hypothesised that BAG3 may interact with either EGFR or components of the EGFR downstream pathways. We firstly examined EGFR expression in TNBC cell lines (*n* = 8). In three TNBC cell lines expressing high levels of BAG3 we observed high expression and activation of EGFR (MDA-MB-468, BT-549 and HCC1143) (Figure 4A). In three TNBC cell lines exhibiting low expression of BAG3 we observed low expression and activation of EGFR (HCC38, HCC70, HCC1937) (Figure 4A). The greatest increase in EGFR expression and activation was observed in MDA-MB-468 cells. Using Pearson’s correlation analysis, we calculated a correlation score of R= 0.7256 (*P* < 0.05) between BAG3 and EGFR expression in the eight TNBC cell lines examined (Figure 4B). We also observed expression of EGFR in a small number of patient tissues expressing BAG3 (*n* = 10). (Supplementary Figure 6).

We hypothesised that BAG3 may regulate tumour cell proliferation by regulating EGFR signalling networks. In order to determine if BAG3 interacted with EGFR signalling components we used a proteomics approach. We explored the BAG3 protein interactome in BT-549 and MDA-MB-468 cells by mass spectrometry. IgG controls were included and non-specific proteins subtracted to generate final lists. A total of 1171 and 1260 protein were identified in BAG3 immunoprecipitates (IPs) from MDA-MB-468 and BT-549 cells (*n* = 2) (Supplementary Table 1). We identified a total of 57 and 31 peptides unique to BAG3 in BT-549 and MDA-MB- 468 cells by mass spectrometry confirming successful target enrichment and the high abundance of BAG3 in the IPs was further confirmed by immunoblotting (Figure 4C). The BAG3 interacting proteins in both cell lines were further analysed by Panther Pathway Analysis and the top 8 signalling pathways associated with BAG3 (10 + genes) are displayed in Figure 4D. This included the ubiquitination proteasome pathway, EGF and FGF signalling pathways. We further analysed the EGF pathway for the BAG3 interacting proteins that form part of this pathway in both BT-549 and MDA-MB-468 cells. These proteins are displayed in pie charts and include EGFR, PLCγ, Extracellular signal related kinase (ERK), proto oncogenes cRAF, Signal transducer and activator of transcription (STAT1) (Figure 4E). Some of these proteins are not highly abundant in the proteomic analysis as expected due to the low copy number of kinases in the cell. Therefore we confirmed selected interactions by immunoblotting new BAG3 IPs. We also probed for Src which was identified by mass spectrometry and is a known EGFR interacting protein but not listed under EGF signalling in the Panther Pathway maps (Figure 4F and 4G). These datasets strongly suggest a novel association between BAG3 with EGFR signalling nodes in TNBC. In order to determine if EGFR may be regulated by BAG3, we measured EGFR expression by densitometry of EGFR immunoblots in MDA-MB-468 cells treated with siBAG3. A small but significant decrease in EGFR protein expression was observed in this cell line (0.81 ± 0.03) compared to the control (1 ± 0.07) (Figure 4H, 4I). To examine if BAG3 may influence activation of EGFR, we examined activation of EGFR after gene silencing of BAG3 using antibodies targeting pEGFRTyr1173, pEGFRTyr1069 and pEGFRpTyr1110. A significant decrease in pEGFRTyr1110 was observed after gene silencing of BAG3 (Supplementary Figure 7).

### Silencing BAG3 reduces activation of the AKT and FAK signalling pathways which regulate proliferation in TNBC cell lines

**Figure 5:**
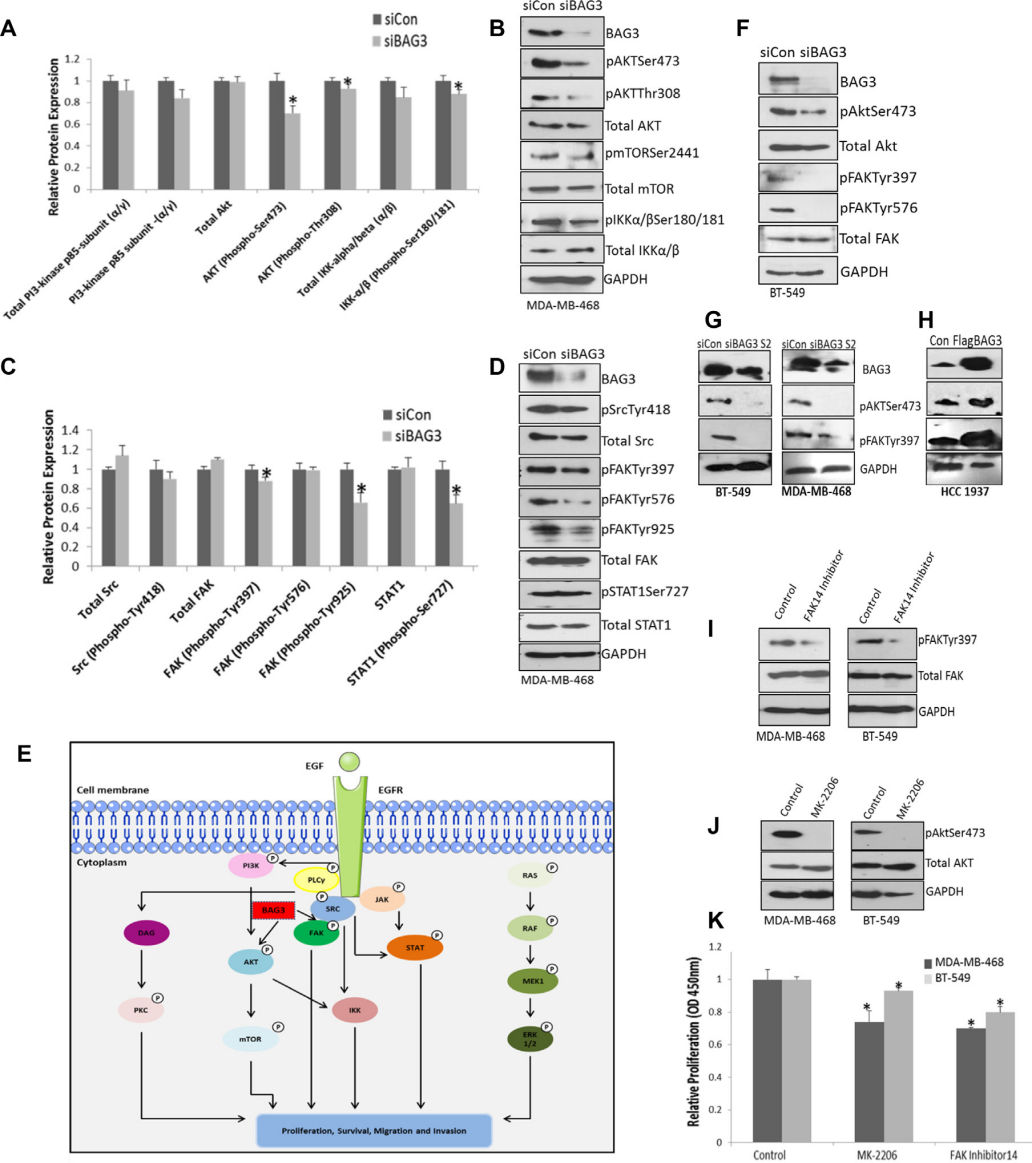
Silencing BAG3 reduces activation of the AKT and FAK signalling pathways which regulate proliferation in TNBC cell lines (**A**) MDA-MB-468 cells were treated with siControl or siBAG3 and EGF pathway analysis performed on lysates using an EGF phospho antibody array. Quantitative histograms of PI3K/AKT signalling components from the EGF array are displayed. The histograms represent average protein expression (± SD) (*n* = 6). (**B**) The protein expression of pAktSer473, pAktThr308, pmTORSer2441, pIKKα/βSer180/181, Akt, mTOR, IKKα/β and BAG3 was confirmed by immunoblotting. (**C**) Quantitative histograms of FAK/Src pathway components. The histograms represent average protein expression (± SD) (*n* = 6). (**D**) The protein expression of pSrcTyr418, Src, pFAKTyr397, pFAKTyr576, pFAKTyr925, FAK, pSTAT1Ser727, STAT1 was confirmed by immunoblotting. (**E**) A hypothetical model of potential regulation of EGFR downstream signaling pathways by BAG3. The diagram was produced using Servier Medical Art (**F**) The protein expression of pFAKTyr397, FAK, pAKTSer473, AKT in BT-549 cells treated with siBAG3 and siControl was confirmed by immunoblotting. (**G**) The protein expression of pFAKTyr397, pAKTSer473 and GAPDH in MDA-MB-468 and BT-549 cell lines treated with an additional siRNA sequence (S2) targeting BAG3 and was confirmed by immunoblotting. (**H**) The protein expression of pFAKTyr397, pAKTSer473 and GAPDH in HCC1937 cells treated with FlagBAG3 and siControl was confirmed by immunoblotting. (**I**) BT-549 and MDA-MB-468 cells were treated with 5 uM MK-2206 and FAK14. Reduced activation of pFAKTyr397 after FAK14 treatment was confirmed by immunoblotting. (**J**) Reduced activation of pAKTSer473 after MK-2206 treatment was confirmed by immunoblotting. (**K**) A quantitative graph of proliferation relative to the control in MDA-MB-468, and BT-549 cells after treating with FAK and MK-2206 inhibitors. The histograms represent mean ( ± SD) Brdu incorporation relative to the control (*n* = 3). An asterisk represents *p* < 0.05.

Given the positive association identified between BAG3 and EGFR signalling nodes identified in Figure 4, we hypothesised that inhibiting BAG3 may result in altered activation of specific EGFR modules or subnetworks. In order to examine this, we employed a second omics type approach an EGF pathway phospho antibody array which featured 214 antibodies related to the EGF pathway. We compared protein expression and phosphorylation status in EGFR signalling pathways in MDA-MB-468 cells treated with siControl and siBAG3. The intensity values for all EGF phosphoproteins before and after BAG3 silencing is listed in Supplementary Table 2. In this figure we highlight the regulation of specific EGFR signalling subnetworks by BAG3 including PI3K/AKT and FAK/SRC and Raf-MEK-ERK, mediated signalling pathways. Analysis of downstream EGF signalling PI3K/AKT pathway revealed a significant decrease in the activation of specific signalling components in this pathway including pAkt-Thr308 and pAkt-Ser473 when BAG3 was gene silenced (Figure 5A). Further downstream of AKT there was a decrease in activation of pIKK(*α*/*β*)-Ser180/181 relative to the control. Interestingly, a significant decrease was also observed in the activation of FAK signaling upon treatment with siBAG3 (pFAK-Tyr925, Tyr397) (Figure 5C). Alterations in activation of both the AKT and FAK pathways were confirmed by immunoblotting (Figure 5B, 5D). Generally, we observed positive validation of the changes in EGF signalling pathways by immunoblotting. However, there was some discordance between the expression of certain phosphoproteins (e.g. pFAKTyr596) validated by this method. We believe this is because of the differences in techniques used. Denatured conditions were used for immunoblotting whereas antibody proteins immobilized on the EGF array are not denatured and have native tertiary structures which can lead to inaccessible target epitopes on the protein in some instances. Activation of RAF/MEK/ERK was also examined. There was a decrease in pMEKSer221 and pERKTyr204 activation using phosphoarray analysis after BAG3 silencing. However, we were not able to confirm the pERK reduction by immunoblotting, possibly because the antibody tested for validation does not differentiate between pERK (Tyr202 and Thr204) (Supplementary Figure 8). These results indicate that BAG3 could be an important regulator of specific EGFR signalling subnetworks. A hypothetical model of BAG3 regulation of the highlighted signalling networks is displayed in Figure 5E. Activation of AKT and FAK was further validated in another TNBC cell line, BT-549 cells after treatments with siControl and siBAG3. A decrease in activation of pAKT-Ser473 and pFAK-Tyr-397 was observed (Figure 5F). Additionally, another siRNA targeting BAG3 (S2) was tested in both BT-549 and MDA-MB-468 cell lines and a reduction in the activation of AKT and FAK pathways was once again observed (Figure 5G). An increase in activation of AKT and FAK was also observed when BAG3 was overexpressed in HCC1937 cells. (Figure 5H). To functionally link activation of the AKT and FAK signalling modules to the regulation of cellular proliferation in TNBC, we explored if inhibiting these pathways would contribute to a reduction in proliferation in TNBC cells. Inhibition of FAK and AKT activation by the chosen inhibitors was confirmed by immunoblotting (Figure 5I, 5J) and encouragingly a significant reduction in proliferation in both cell lines was observed, (Figure 5K). In addition to employing FAK/AKT inhibitors to target these pathways, we also gene silenced AKT1 an FAK1 (to increase specificity) and measured proliferation. A significant decrease in proliferation was observed again when AKT1 and FAK1 were gene silenced (Supplementary Figure 9). These results suggest that BAG3 may regulate proliferation through the AKT and FAK signalling modules in the TNBC cell lines tested.

### Targeting BAG3 with siRNA or protein interaction inhibitors reduces cell proliferation and viability in TNBC cell lines

**Figure 6:**
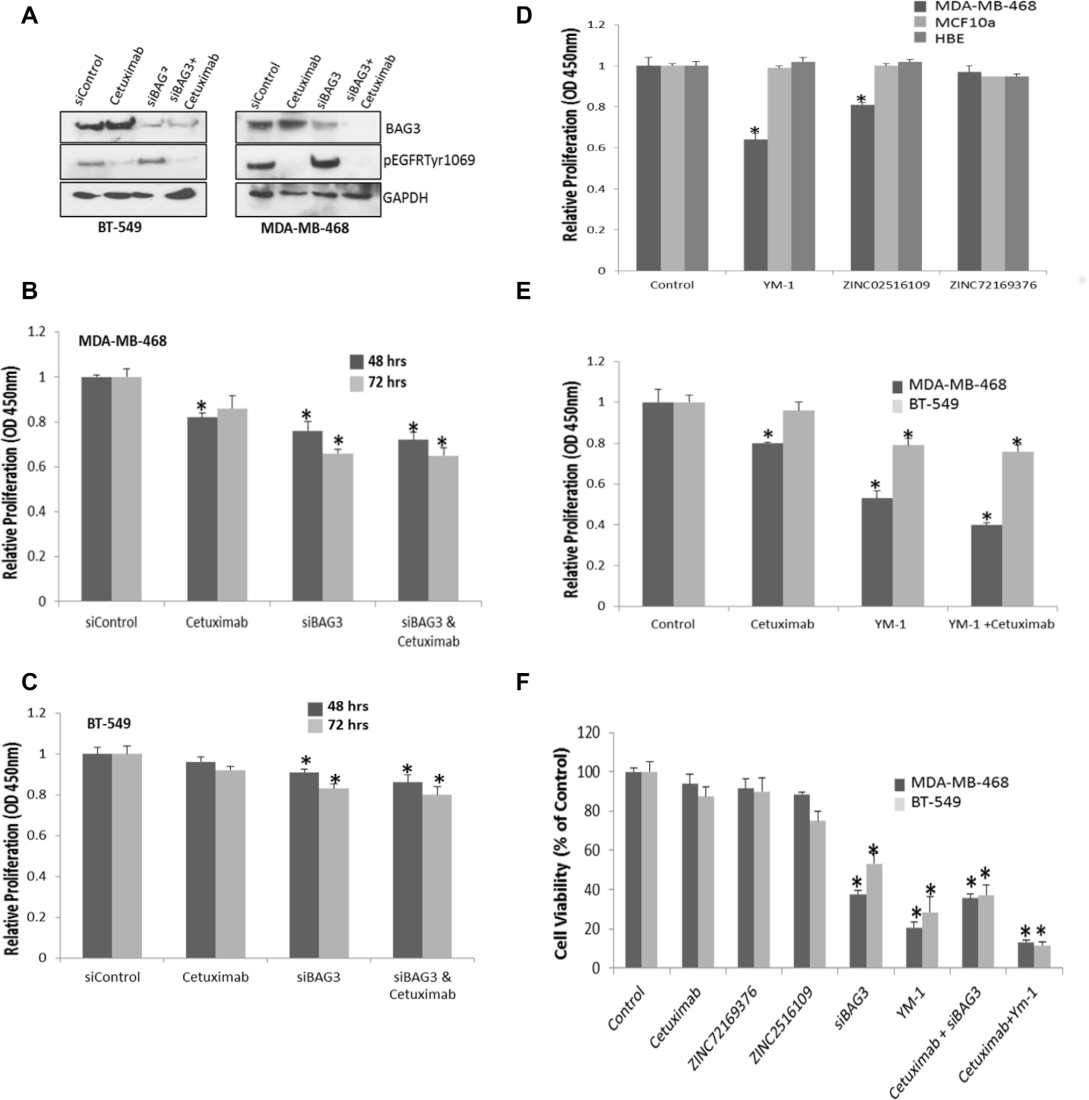
Targeting BAG3 with siRNA or protein interaction inhibitors reduces cell proliferation and viability in TNBC cell lines (**A**) BT-549 and MDA-MB-468 cells were treated with either siBAG3, siControl or treated with 25 µg/mol Cetuximab for 48 hrs. Reduced activation of EGFR (pEGFRTyr1069) and expression of BAG3 was confirmed by immunoblotting. (**B**) A quantitative graph of proliferation in MDA-MB-468, and (**C**) BT-549 cells after treating with siControl, Cetuximab, siBAG3 and siBAG3 combined with Cetuximab for 48 and 72 hrs. The histograms represent mean (± SD) Brdu incorporation relative to the control (*n* = 3). An asterisk represents *p* < 0.05 (**D**) A quantitative graph of proliferation in MDA-MB-468, MCF10a and HBE cells after treating with a DMSO Control, 5 uM YM-1, 10 uM ZINC02516109 and ZINC72169376 compounds.The histograms represent mean (± SD) Brdu incorporation relative to the control (*n* = 3). An asterisk represents *p* < 0.05. (**E**) A quantitative graph of proliferation in MDA-MB-468 and BT-549 cells after treating with siControl, Cetuximab and YM-1 combined with Cetuximab. The histograms represent mean (± SD) Brdu incorporation relative to the control (*n* = 3). An asterisk represents *p* < 0.05. (**F**) MDA-MB-468 and BT-549 cells were treated with siBAG3, 25 ug/ mol Cetuximab, 5 uM YM-1, 10 uM ZINC02516109 and 10 uM ZINC72169376. Cell viability was measured using an MTT assay. A quantitative graph of average cell viability relative to the control is displayed (*n* = 3). An asterisk represents *p* < 0.05.

As BAG3 influences the regulation of EGFR signal transduction networks which could be rewired following traditional EGFR therapy, we postulated targeting BAG3 alone or in combination with EGFR may be beneficial to TNBC patients. We tested this hypothesis in TNBC cell lines MDA-MB-468 and BT-549 cells treated with both the EGFR inhibitor Cetuximab and siBAG3. Gene silencing of BAG3 and reduction of EGFR phosphorylation after Cetuximab was confirmed by immunoblotting (Figure 6A). A significant decrease in proliferation was observed after treating MDA-MB-468 and BT-549 (*p* < 0.05) cells with siBAG3 and siBAG3/Cetuximab relative to the control at 48 hrs and 72 hrs (Figure 6B, 6C) respectively although combined treatment compared to treatment with siBAG3 alone was additive not synergistic. As an alternative to gene silencing, we investigated if we could identify BAG3 protein interaction inhibitors using a high throughput *in silico* screening analysis tool. To design protein-protein interaction (PPI) inhibitors we used a combination of homology modelling and compound screening. We identified different compounds that could bind the HSP70-BAG3 virtual interface at the designated pharmacore points. The compounds called carbamimidamido-2-[2-(phenylformamido) acetamido]pentanoicacid (ZN02516109) and (3-(4,5-Dimethylthiazol-2-yl)-2,5-Diphenyltetrazolium Bromide) acid (ZINC72169376) which bound 4 pharmacore points (Supplementary Figure 10) at the HSP70-BAG3 interface were tested in addition to YM-1 a compound reported to disrupt the BAG3:HSP70 interaction in breast cancer cell lines [13]. YM-1 does not deplete BAG3 but disrupts its interaction with HSP70 (Supplementary Figure 11A) which is important in the proteasomal regulation of key signalling molecules. A significant decrease in proliferation in a TNBC cell line relative to the control cell lines was observed with compounds ZN02516109, however inhibition with YM-1 showed a greater reduction of proliferation (0.67 ± 0.01) versus (0.85 ± 0.01) for ZN02516109 (Figure 6D). We also observed decreased activation of the AKT and FAK pathways after YM-1 treatment mirroring what we observed with BAG3 gene silencing (Supplementary Figure 11B). Therefore in this study we tested YM-1 alone and in combination with Cetuximab. A significant decrease in proliferation was observed using YM-1 and YM-1/Cetuximab combined, compared to the control. The combined effects of YM-1 with Cetuximab were synergistic in MDA-MB-468 cells and additive in BT-549 cells. These results suggest that knockdown of BAG3 by gene silencing or disruption of its interactions by PPI inhibitors could improve the TNBC response compared to the control (Figure 6E). Additionally a greater decrease in cell viability was observed using YM-1/Cetuximab or BAG3/Cetuximab compared to either the control or Cetuximab alone with a synergistic effect again being observed in MDA-MB-468 cells (Figure 6F). This was performed at 72 hrs to demonstrate impact of targeting BAG3 at a longer timepoint.

## DISCUSSION

Triple-negative breast cancers are aggressive cancers, with a significant risk of disease recurrence in the first 5 years following diagnosis. There are no established targeted therapeutics or biomarkers response clinically approved in the context of TNBC [2]. We have observed high expression of BAG3 in ∼50% of TNBC cell lines and patient samples. Furthermore, BAG3 expression in TNBC patients derived from multiple clinical datasets [24, 25] showed high mRNA expression of BAG3 significantly correlates with lower recurrence free survival. High BAG3 expression has previously been associated with a worse prognosis and poorer survival in aggressive cancers such as pancreatic adenocarcinoma [16] medullablastoma [26] and metastatic melanoma [27]. These initial studies suggest a potential role for BAG3 as a prognostic marker of disease recurrence in TNBC although further studies are required. Due to the high expression of BAG3 observed in a subset of TNBC cell lines and patient samples, we hypothesised that BAG3 might regulate tumour cell proliferation. BAG3 has been reported to sustain proliferation [18], migration [19] and invasion [20] in diverse cancer subtypes including colorectal cancer ovarian and hepatocellular cancer subtypes. Encouragingly, gene silencing of BAG3 led to a decrease in cell proliferation and migration in the TNBC cells in this study. The ability of BAG3 to exert a pro-survival role in TNBC cells suggested that targeting BAG3 may be useful in TNBC certain patients.

In order to further stratify which patients may benefit from targeting BAG3, we examined EGFR expression in a panel of TNBC cell lines. EGFR is amplified in TNBC patients [4] and we observed a significant correlation between BAG3 and EGFR expression in a panel of TNBC cell lines. The multimodal protein domain structure of BAG3 allows many interactions with intracellular signalling molecules [10]. Our previous work demonstrated a role for BAG3 in regulation of the ERK signalling pathway in different breast cancer cell models [28]. This led us to hypothesise a role for BAG3 in modulation of EGFR signal transduction networks that may in turn influence cellular proliferation in TNBC. Gene expression enrichment studies have revealed both EGF signalling pathway and downstream signalling targets such as the PI3K/AKT to be activated in different TNBC subtypes [29, 30]. In this study we used systems based omics approaches to investigate BAG3 regulation of EGFR signalling networks. We used mass spectrometry based proteomics to determine if BAG3 interacted with EGFR signalling components and antibody array technology to investigate which signalling modules were altered upon BAG3 silencing. Pathway Analysis revealed EGFR signalling to be enriched in the BAG3 interactome of TNBC cell lines validating our hypothesis. Using this proteomics approach, we discovered that BAG3 associates with EGFR as well as Raf1, PLCϒ, ERK, STAT1 and Src. These findings support earlier studies showing a previous interaction between BAG3 and the SH3 domain of Src [13] and PLCϒ [12]. To determine if there is any regulation of EGFR by BAG3, we reduced BAG3 expression by gene silencing and observed a small but significant decrease in EGFR expression. Further analysis into regulation of EGFR by BAG3 revealed significant reduction in expression of pEGFRTyr1110 but not additional activation sites such as pEGFRTyr1173. A recent study showing Src kinase which normally phosphorylates EGFR at tyrosyl 1173, could mediate phosphorylation of Tyr1110 in EGFR, and regulate mitogenic signalling [31]. Therefore BAG3 could possibly regulate pEGFRTyr1110 through it’s interaction with Src kinase. Further investigation into this mechanism would be required. Given the prevalence of secondary mutations in TNBC patients such as PTEN loss and BRAF mutants [32] we were particularly interested whether BAG3 could influence downstream EGF signalling networks.

Deeper investigation into which signalling subnetworks maybe influenced by BAG3 was obtained from analysis of the EGF phospho signalling networks. Remarkably, we observed that inhibiting BAG3 resulted in reduced activation both of the AKT and FAK signalling pathways. We observed a reduction in AKT activation and a decrease in activation of downstream AKT target, pIKKα/β which has been previously reported to be stabilised by BAG3 and regulate tumour cell proliferation [33]. We did not identify an interaction between BAG3 and AKT by mass spectrometry but suggest that BAG3 directly could regulate AKT stability by preventing Hsp70 mediated proteasomal degradation of this client protein as reported in a previous study [34]. We also identified a reduction in activation in the FAK pathways. BAG3 may regulate FAK phosphorylation indirectly though it’s interaction with Src [13]. A role for Src-induced tyrosine phosphorylation of FAK in survival and growth of transformed cells has previously been described [35]. We hypothesised that BAG3 may regulate proliferation in TNBC cells via the AKT or FAK mediated signalling pathways. Our data demonstrated a decrease in proliferation in TNBC cell models using siRNAs and inhibitors to FAK and AKT suggesting that this is a plausible mechanism.

Importantly, activation of downstream bypass signalling pathways such as PI3K/AKT through additional mutations such as PTEN loss are possible mechanisms of resistance to current EGFR antibody treatments such as Cetuximab which are not proving successful as single therapies in TNBC patients in the clinic [36]. Carey *et al.* examined Cetuximab in a metastatic advanced recurrent breast cancer clinical trial. Cetuximab failed to inhibit EGFR signaling in 72% (13 of 18) of the patients, suggesting alternative pathways may be present [37]. Additionally, several TNBC cell lines have shown to be unresponsive to Cetuximab [38]. We hypothesised that targeting by gene silencing BAG3 may improve therapeutic response compared to Cetuximab alone as BAG3 can reduce activation of several pathways in TNBC cell lines. We did identify improved therapeutic response after genetic silencing of BAG3 but the addition of Cetuximab was not beneficial. This may be in part, due to the fact the TNBC cell lines tested, were not very responsive to Cetuximab as reported in previous studies [38]. As an alternative to inhibiting BAG3 by gene silencing, we use an *in silico* approach to investigate novel protein interaction inhibitors targeting the BAG3/HSP70 interaction. A crystal structure of the BAG3: HSP70 interaction is not currently available therefore we employed a homology modelling approach and screened for molecules that docked to the BAG3-HSP70 binding site. Successful ligand discovery from a modelled structure of G Protein Receptors has previously been reported [39]. Two compounds were identified using this approach and their efficacy in reducing proliferation was investigated in TNBC cells. Although one compound identified in the screen caused a significant reduction in proliferation (ZN02516109), YM-1 a HSP70 allosteric inhibitor shown to target HSP70:BAG3 [13] was more effective at reducing cell proliferation and viability in TNBC cells. Of note, YM-1 targeted the same pathways as BAG3 in this study. Reduced activation of FAK and AKT was observed with Ym-1 (Supplementary Figure 11B). As mentioned silencing of BAG3 can potentially destabilise AKT by increasing it’s proteasomal degradation [34]. YM-1 similarly has been reported to increase client protein ubiquitination and degradation [40]. We are currently performing further studies to characterise novel BAG3 protein interaction inhibitors that could be beneficial in TNBC.

In this study we describe a novel role for BAG3 in driving tumour proliferation in TNBC by stabilising EGFR signalling transduction pathways. Signal transduction networks are often rewired in cancer cells and identifying therapies that can target more than one pathway may enable more effective cancer treatment. The ability of BAG3 to regulate several signalling modules simultaneously makes it an attractive therapeutic target.

## MATERIALS AND METHODS

### Reagents

Cell culture medium was purchased from Invitrogen (Grand Island, NY). Fetal bovine serum (FBS) was purchased from Atlas Biologicals (Fort Collins, CO). Primary antibodies targeting the following were as detailed: Actin, EGFR, pEGFRTyr1069, pEGFRTyr1173, pEGFRTyr1110, ERK1/2, pERK1/2Thr202/204, mTOR, pmTORSer2441, pFAKTyr397, pFAKTyr527, pFAKTyr925, FAK, pMEKSer221, MEK, IKKα/β, pIKK α/βSer180/181, pSRCTyr418, SRC, pAKTSer473, AKT, pSTAT1Ser727, STAT1, Hsp70 and PLCγ were from Cell Signalling (Danvers, MA). Antibodies specific for BAG3 were from Abcam (Cambridge, UK). Secondary HRP-conjugated antibodies, including anti-rabbit Gig and anti-mouse IgG were from Cell Signalling (Danvers, MA). YM-1 was obtained from Sigma Aldrich and the compounds 5-carbamimidamido-2-[2-(phenylformamido) acetamido pentanoic acid (ZINC02516109) and 2-(quinoline-3-carbonyl)-2,8-diazaspiro[4.5]decane-3-carboxylic acid (ZINC72169376) sourced from MolPort (Europe).

### Cell culture

MDA-MB-231 and MDA-MB-468 were cultured in Dulbecco’s modified Eagle’s medium (DMEM) supplemented with 10% FBS and 5 mM glutamine. HCC1143, HCC1937 and BT-549 cells HCC38 were cultured in RPMI-1640 supplemented with 10% FBS and 1% Sodium Pyruvate. MDA-MB-436 cells were cultured in Leibovitz’s L-15 Medium supplemented with 10% FBS and 5 mM glutamine. MCF 10a and 184B5 cell lines were cultured in DMEM and supplemented with the following; Horse serum 25.0 ml (5% final) EGF 100 µl (100 µg/ml stock) (20 ng/ml final) Hydrocortisone 250 µl (1 mg/ml) (0.5 µg/ml final) Cholera toxin 5 µl (1 mg/ml stock) (100 ng/ml final). Insulin 500 µl (10 mg/ml stock) (10 µg/ml final). Cells were grown in humidified cell culture incubators under 5% CO_2_, 95% air. All cells were stimulated with 5 ng/ml hEGF in medium for 30 minutes at 37° C before cells lysis or assay end point.

### RNA Isolation

Total RNA was extracted from tissue samples with TRIzol (Invitrogen). cDNA was synthesised using an iScript synthesis kit (Bio-Rad). The complete reaction mix was incubated for 5 minutes at 25°C, 30 minutes at 42°C and 5 minutes at 85°C. RT-PCR was performed by the Genomic Core facilities at UCD, using iQSYBR Green Supermix (Bio-Rad). Real time reactions were performed on a Bio-Rad iCycler. Real time PCR primers were as follows: BAG3, forward 5′CAACAGCCGCACCACTAC-3′; reverse, 5′-CATTGGCAGAGGATGGAGTC-3′ GAPDH was employed to normalise the expression of the target gene BAG3.

### Protein extraction and immunoblotting

Cell extracts were prepared in IP lysis buffer (0.25 M Tris, 0.15 M NaCl, 1 mM EDTA, 1% NP-40, 5% glycerol, mammalian protease inhibitor cocktail, pH 7.4). Extracts were centrifuged at 16,000 × g for 10 minutes and stored at –80° C. Protein concentration was determined by the BCA assay (Thermo Scientific, Rockford, IL). For Western blotting, equal amounts of protein were resolved by SDS-PAGE and transferred onto a 0.2 mm nitrocellulose membrane. Membranes were blocked (50 mM Tris, pH 7.6, 150 mM NaCl, 0.05% Tween 20, 5% nonfat dry milk or BSA) prior to incubation with antibodies. Luminol-based detection was performed using SuperSignal West Pico or Femto reagents (Thermo Scientific, Rockford, IL).

### Public TNBC transcriptomic datasets

An *in-silico* method was adopted to explore publicly available TNBC datasets in which a method for assigning TNBC status to transcriptomic data from human breast cancer tissues was employed. A TNBC microarray dataset of 383 patients was analysed to identify if BAG3 was associated with disease free survival [24]. This entire TNBC microarray dataset was derived from patients with a median age of 50 years (range 28–88 years) at the time of diagnosis, and a median follow-up of 51 months (range 0–10 years). TNBC patient samples were defined on the basis of negative mRNA expression of *ER, PR*, and *HER2* genes (Cohort 1). Kaplan Meier survival analysis with Log-rank (Mantel-Cox) testing was employed to investigate if the BAG3 gene was significantly associated with recurrence free survival in this dataset. As a second approach the online tool, *BreastMark*, was used to analyse an association between BAG3 and patient disease free survival using the *BreastMark* resource as previously described [25]. In this study, *BAG3* mRNA expression data was analysed from 2,656 breast cancer patients of mixed subtypes, and then in a subset of 309 patients identified by custom analysis which filtered TNBC patient samples based on negative mRNA expression of the *ER, PR* and *HER2* genes (Cohort 2). Survival curves based on Kaplan-Meier estimates were used to determine the relationship between BAG3 and survival and recurrence free survival in this Cohort. The log rank *p*-value was calculated to determine significance. Cox regression analysis was used to calculate hazard ratios in all Cohorts.

### IHC and tissue analysis

Full tissue sections were obtained from a cohort of 80 patients (Cohort 3) diagnosed with Triple Negative Breast Cancer at St. Vincent’s University Hospital, Dublin Ireland between 2000 and 2010 who had not received chemotherapy. All the patients provided an informed consent for using their samples. In this study, patients were censored at 10 years with a median age of 67 years (range between 32–99 years).

Deparaffinisation, antigen retrieval and IHC staining for BAG3 and EGFR were performed on an automated platform (Bond™ III system – Leica MicroSystems™, Newcastle, U.K.). Staining for BAG3 was performed using a rabbit monoclonal antibody (anti- BAG3 Abcam cat# ab92309; 1:300 dilution, ER1 antigen retrieval for 20 minutes). For EGFR immunostaining, a mouse monoclonal antibody (Leica Biosystems NCL-L-EGFR-384; 1:100 dilution, ER1 antigen retrieval for 20 minutes) was used. Antibody optimisation was performed on invasive breast cancer (BAG3) and placental (EGFR) TMAs.

Histopathological review of the sections was performed for BAG3 and the tissues were graded initially as (0, 1, 2, 3) and further graded as either high BAG3 expressing tissues (Score 2, 3) or as low BAG3 (Score 0, 1) expressing tissues. Survival curves based on Kaplan-Meier estimates were used to determine the relationship between BAG3 and recurrence free survival in this Cohort (Cohort 3).

### siRNA transfections

Cells were transfected with 5 nM siRNA BAG3 using 4 µl Lipofectamine (Invitrogen, Grand Island, NY) as described previously [28]. Briefly, lipid-siRNA complexes were prepared in serum-free media and added to cell suspensions in culture medium with 10% FCS. After 24 hours, the medium was replaced. The target sequences were as follows BAG3: S1: 5′-GCCUGAAAACAAACCAGAATT-3′ and S2: 5-GCCAUUGAUGUCCCAGGUCTT-3, AKT 1: 5′-GCGUGACCAUGAACGAGUUTT-3′ and 5′-CGGUAGCACUUGACCUUUUTT-3. FAK 1; 5′ GAUGUUGGUUUAAAGCGAUTT-3′ and 5′-CGAUAUAUGGAAGAUAGUATT-3′. Cellular assays and protein analysis was performed at 48–72 hrs post-transfection.

### Plasmid constructs and co-transfection

The construct for Flag-Bag3 was previously reported [28]. Cells were transfected using 10 μg of either the expression construct or empty vector using Lipofectamine 2000.

### Migration assay

Cell migration was assessed with a wound healing scratch assay. Cells were cultured until confluent in a 6-well culture plate. 24 hours post-transfection, a scratch was made on the confluent monolayer using a sterile 200 µl tip. Cells were washed in warm DPBS to remove any detached cells. Hereafter, cells were photographed at *t* = 0 and *t* = 24 hours with a microscope coupled to a digital camera (QIMAGING). Cell migratory rates were quantified in triplicate using Q-capture-pro-7 software and percentage wound closure was calculated. (T0-T24/T0) ×100.

### Immunoflourescence

Breast cancer cell lines (BT-549 and MDA-MB-231) were seeded at 50,000 cells per 96 well, left overnight and scratch wounds were made using sterile p10 pipette tips. Wounds were allowed to heal for 24 hrs before being fixed using 4% PFA. Actin was stained using AlexaFluor568 labelled phalloidin and Hoechst 33342 was used to visualise the nuclei. Images were acquired using Leica DMI6000 microscope with a 10X objective. Scale bar = 50 µm.

### Invasion assay

Using a BME Cell Invasion assay (R&D Systems, UK), a 96-well Boyden chamber with 8 µm pore size polycarbonate membrane was pre-coated with matrigel to form a matrix barrier. An aliquot of 1 × 10^4^ cells transfected for 24 hours, were seeded with serum free media in the upper chamber. Media supplemented with 10% serum (as a chemoattractant) added to the lower chamber. Cells were allowed to invade for 24 hours and then dissociated from the reverse side of the membrane. Dissociated cells were transferred to a gridded 96 well plate with 9 fields and incubated with poly-L-lysine before fixed in 4% PFA and stained with DAPI. All invaded cells were imaged using a fluorescent microscope (ZEISS Axiovert 200M) coupled to an Axiovision digital camera and counted using ImageJ.

### Proliferation assay

Cell proliferation was assessed using a Using BrdU cell proliferation colorimetric ELISA kit (Abcam, Cambridge) as per the manufacturer’s instructions. Samples were measured using a SpectraMaxM3 plate reader at OD450/540 nm. Samples were run in triplicate.

### MTT-cell viability assay

MDA-MB-468 and BT-549 cells were seeded in a 96-well plate at 5 × 10^3^ cells per well. Cells were left to adhere overnight. Cells were incubated at 37° C with vehicle control or treatment for a time of 48 hours. 40 µl of 2.5 mg of MTT reagent (3-(4,5-Dimethylthiazol-2-yl)-2,5-Diphenyltetrazolium Bromide) dissolved per 1 mL of PBS) was added directly to the cell culture medium and incubated for 2 hours at 37° C or until a sufficient crystal formation in the cells was observed. Media was removed and 100 mL of DMSO was added to each well to lyse the cells. The plate was shaken briefly and the absorbance was read at 595 nm on a Versamax plate reader.

### Cell death assays

Cell death was quantitatively assayed by antibody-mediated capture and detection of cytoplasmic mononucleosome-associated histone-DNA complexes using the Cell Death Detection ELISA Plus kit from Roche Diagnostics (Indianopolis, IN) as per manufacturer’s instructions.

### EGF pathway phosphorylation antibody array

Activation or EGFR pathways was assessed using an EGF pathway phospho antibody array which featured 214 antibodies related to the EGF pathway (Full Moon, Biosystems, CA), cells were treated with siControl or siBAG3 constructs and stimulated with EGF ligand before following the manufacturer’s instructions. Slides were coupled with a Cy3 fluorescent dye. Array slides were imaged on an Axon GenePix 4000GB and quantified with GenePix pro software.

### Endogenous IP

Cell extracts were prepared in IP lysis buffer and clarified by centrifugation (16,000 × g,10 min). BAG3 was crosslinked with Protein A/G Magnetic Dynabeads (Thermo Scientific, Rockford, IL) using a DMP crosslinking protocol. The crosslinked antibodies were then incubated with cell extracts (5 mg of protein) overnight at 4° C. Beads were washed three times with IP lysis buffer and eluted in urea for MS analysis.

### Proteomic sample preparation

The endogenous immunoprecipitates were resuspended in 8M urea. The samples were reduced by incubation for 20 minutes with 5 mM tris (2-carboxyethyl) phosphine at room temperature and alkylated in light exclusion by treatment with 10 mM iodoacetamide for 15 minutes. Proteins were digested overnight at 37° C with Sequencing Grade Modified Trypsin (Promega, Madison, WI, USA). Proteolysis was stopped by acidification.

### Mass spectrometry

Protein digests were pressure-loaded into 250-μm i.d capillaries packed with 2.5 cm of 10-μm Jupiter C18 resin (Phenomenex). Endogenous IP samples were run on a Thermo Scientific Q Exactive mass spectrometer connected to a Dionex Ultimate 3000 (RSLCnano) chromatography system. Tryptic peptides were re-suspended in 0.1% formic acid. Each sample was loaded onto a Biobasic Picotip Emitter (120 mm length, 75 μm ID) packed with Reprocil Pur C18 (1.9 μm) reverse phase media and was separated by an increasing acetonitrile gradient over 60 min at a flow rate of 250 nL/minute. The mass spectrometer was operated in positive ion mode with a capillary temperature of 220° C, and with a potential of 2100V applied to the frit. A top 12 method was used. Full MS scans were acquired in the Orbitrap mass analyser over the range m/z 300–1600 with a mass resolution of 70 000 (at m/z 200). The target value was 3.00E + 06. The twelve most intense peaks with were fragmented in the HCD collision cell with a normalized collision energy of 27%, and tandem mass spectra were acquired in the Orbitrap mass analyser with a mass resolution of 17500 at m/z 200.

### Analysis of tandem mass spectra

The Q-exactive raw data files were *de novo* sequenced and cross searched against a Human UniProtKB database Release 2013_07, 20,266 entries using the search engine PEAKS Studio 7, for peptides cleaved with trypsin. Each peptide used for protein identification met specific Peaks parameters, that is, only peptide scores that corresponded to a false discovery rate (FDR) of ≤1% were accepted from the Peaks database search. The database searching parameters included up to two missed cleavages allowed for full tryptic digestion, and a precursor ion mass tolerance 10 ppm. A fixed modification of cysteine 57.02146 was included and variable modifications included up to 707 common modifications for the Peaks PTM search.

### Panther and ingenuity analysis

Proteins identified by mass spectrometry were further analysed by The PANTHER (Protein ANalysis THrough Evolutionary Relationships) Classification System which was designed to classify proteins (and their genes) in order to facilitate high-throughput analysis http://pantherdb.org/. Proteins were classified according to the Panther Pathway tool. The PANTHER Classifications are the result of human curation and bioinformatics algorithms (Hidden Markov Models). Details of the methods can be found as previously described [41].

### Homology modelling/PPI compound screen

To design protein-protein interaction (PPI) inhibitors we used a combination of homology modelling and compound screening as previously described. Both structure based drug discovery methods are dependent on a 3-dimensional target structure. As the Hsp70-Bag3co-complex has not yet been crystallised, we built a model using MOE software using the structure of the human hsp70-Bag5 complex (3A8Y) as a template. A pharmacophore was designed from the protein-protein interaction interface and used to screen 5 million drug-like commercially available compounds. This resulted in the identification of hit compounds that correctly matched 3–5 pharmacophore points. Available compounds were purchased from Molport and tested using cancer cell proliferation assays as described above.

### Statistical analysis

All data are presented as the mean ± SEM for at least three independent experiments. For each experiment, the statistical tests are indicated in the results section. Student paired *t*-test analysis and Pearson Correlation Analysis was conducted using Prism 5 (GraphPad Software, La Jolla, CA, USA). Quantification of BAG3 expression was performed using Image J densitometry software with BAG3 expression values normalised to the loading control (GAPDH). Kaplan–Meier survival analysis using the Log-rank (Mantel-Cox) test was performed using the SPSS statistical analysis software (IBM). Hazard ratios (HR) and 95% confidence intervals (95% CI) were evaluated using Cox regression analysis.

## SUPPLEMENTARY MATERIALS FIGURES AND TABLES







## Abbreviations

BAG3: Bcl-2-associated athanogene 3
TNBC: Triple Negative Breast Cancer
EGFR: Epidermal Growth Factor Receptor
HER2: Human Epidermal Growth Factor Receptor
ER: Estrogen Receptor
PR: Progesterone Receptor
PI3K: Phosphatidylinositol 3-kinase
mTOR: Mammalian target of rapamycin
ERK: Extracellular signal related kinase
PLCγ: Phospholipase C gamma
FAK: Focal adhesion kinase
PPI: Protein-protein interactors
STAT: Signal transducer and activator of transcription
RGS: Recurrence Free Survival

## References

[1] Collignon J, Lousberg L, Schroeder H, Jerusalem G (2016). Triple-negative breast cancer: treatment challenges and solutions. Breast Cancer (Dove Med Press).

[2] Dent R, Trudeau M, Pritchard KI, Hanna WM, Kahn HK, Sawka CA, Lickley LA, Rawlinson E, Sun P, Narod SA (2007). Triple-negative breast cancer: clinical features and patterns of recurrence. Clin Cancer Res.

[3] Pal SK, Childs BH, Pegram M (2011). Triple negative breast cancer: unmet medical needs. Breast Cancer Res Treat.

[4] Park HS, Jang MH, Kim EJ, Kim HJ, Lee HJ, Kim YJ, Kim JH, Kang E, Kim SW, Kim IA, Park SY (2014). High EGFR gene copy number predicts poor outcome in triple-negative breast cancer. Mod Pathol.

[5] Carey LA, Rugo HS, Marcom PK, Mayer EL, Esteva FJ, Ma CX, Liu MC, Storniolo AM, Rimawi MF, Forero-Torres A, Wolff AC, Hobday TJ, Ivanova A (2012). TBCRC 001: randomized phase II study of cetuximab in combination with carboplatin in stage IV triple-negative breast cancer. J Clin Oncol.

[6] Yi YW, Hong W, Kang HJ, Kim HJ, Zhao W, Wang A, Seong YS, Bae I (2013). Inhibition of the PI3K/AKT pathway potentiates cytotoxicity of EGFR kinase inhibitors in triple-negative breast cancer cells. J Cell Mol Med.

[7] Duncan JS, Whittle MC, Nakamura K, Abell AN, Midland AA, Zawistowski JS, Johnson NL, Granger DA, Jordan NV, Darr DB, Usary J, Kuan PF, Smalley DM (2012). Dynamic reprogramming of the kinome in response to targeted MEK inhibition in triple-negative breast cancer. Cell.

[8] Calderwood SK, Gong J (2012). Molecular chaperones in mammary cancer growth and breast tumor therapy. J Cell Biochem.

[9] Takayama S, Reed JC (2001). Molecular chaperone targeting and regulation by BAG family proteins. Nat Cell Biol.

[10] Behl C (2016). Breaking BAG: The Co-Chaperone BAG3 in Health and Disease. Trends Pharmacol Sci.

[11] Ingham RJ, Colwill K, Howard C, Dettwiler S, Lim CS, Yu J, Hersi K, Raaijmakers J, Gish G, Mbamalu G, Taylor L, Yeung B, Vassilovski G (2005). WW domains provide a platform for the assembly of multiprotein networks. Mol Cell Biol.

[12] Doong H, Price J, Kim YS, Gasbarre C, Probst J, Liotta LA, Blanchette J, Rizzo K, Kohn E (2000). CAIR-1/BAG-3 forms an EGF-regulated ternary complex with phospholipase C-gamma and Hsp70/Hsc70. Oncogene.

[13] Colvin TA, Gabai VL, Gong J, Calderwood SK, Li H, Gummuluru S, Matchuk ON, Smirnova SG, Orlova NV, Zamulaeva IA, Garcia-Marcos M, Li X, Young ZT (2014). Hsp70-Bag3 interactions regulate cancer-related signaling networks. Cancer Res.

[14] Chiappetta G, Basile A, Barbieri A, Falco A, Rosati A, Festa M, Pasquinelli R, Califano D, Palma G, Costanzo R, Barcaroli D, Capunzo M, Franco R (2014). The anti-apoptotic BAG3 protein is expressed in lung carcinomas and regulates small cell lung carcinoma (SCLC) tumor growth. Oncotarget.

[15] Festa M, Del Valle L, Khalili K, Franco R, Scognamiglio G, Graziano V, De Laurenzi V, Turco MC, Rosati A (2011). BAG3 protein is overexpressed in human glioblastoma and is a potential target for therapy. Am J Pathol.

[16] Rosati A, Bersani S, Tavano F, Dalla Pozza E, De Marco M, Palmieri M, De Laurenzi V, Franco R, Scognamiglio G, Palaia R, Fontana A, di Sebastiano P, Donadelli M (2012). Expression of the antiapoptotic protein BAG3 is a feature of pancreatic adenocarcinoma and its overexpression is associated with poorer survival. Am J Pathol.

[17] Iwasaki M, Homma S, Hishiya A, Dolezal SJ, Reed JC, Takayama S (2007). BAG3 regulates motility and adhesion of epithelial cancer cells. Cancer Res.

[18] Shi H, Xu H, Li Z, Zhen Y, Wang B, Huo S, Xiao R, Xu Z (2016). BAG3 regulates cell proliferation, migration, and invasion in human colorectal cancer. Tumour Biol.

[19] Kassis JN, Guancial EA, Doong H, Virador V, Kohn EC (2006). CAIR-1/BAG-3 modulates cell adhesion and migration by downregulating activity of focal adhesion proteins. Exp Cell Res.

[20] Suzuki M, Iwasaki M, Sugio A, Hishiya A, Tanaka R, Endo T, Takayama S, Saito T (2011). BAG3 (BCL2-associated athanogene 3) interacts with MMP-2 to positively regulate invasion by ovarian carcinoma cells. Cancer Lett.

[21] Meng X, Kong DH, Li N, Zong ZH, Liu BQ, Du ZX, Guan Y, Cao L, Wang HQ (2014). Knockdown of BAG3 induces epithelial-mesenchymal transition in thyroid cancer cells through ZEB1 activation. Cell Death Dis.

[22] Xiao H, Cheng S, Tong R, Lv Z, Ding C, Du C, Xie H, Zhou L, Wu J, Zheng S (2014). BAG3 regulates epithelial-mesenchymal transition and angiogenesis in human hepatocellular carcinoma. Lab Invest.

[23] de Sousa Abreu R, Penalva LO, Marcotte EM, Vogel C (2009). Global signatures of protein and mRNA expression levels. Mol Biosyst.

[24] Rody A, Karn T, Liedtke C, Pusztai L, Ruckhaeberle E, Hanker L, Gaetje R, Solbach C, Ahr A, Metzler D, Schmidt M, Muller V, Holtrich U (2011). A clinically relevant gene signature in triple negative and basal-like breast cancer. Breast Cancer Res.

[25] Madden SF, Clarke C, Gaule P, Aherne ST, O’Donovan N, Clynes M, Crown J, Gallagher WM (2013). BreastMark: an integrated approach to mining publicly available transcriptomic datasets relating to breast cancer outcome. Breast Cancer Res.

[26] Yang D, Zhou J, Wang H, Wang Y, Yang G, Zhang Y (2016). High expression of BAG3 predicts a poor prognosis in human medulloblastoma. Tumour Biol.

[27] Guerriero L, Chong K, Franco R, Rosati A, De Caro F, Capunzo M, Turco MC, Hoon DS (2014). BAG3 protein expression in melanoma metastatic lymph nodes correlates with patients’ survival. Cell Death Dis.

[28] Pasillas MP, Shields S, Reilly R, Strnadel J, Behl C, Park R, Yates JR, Klemke R, Gonias SL, Coppinger JA (2015). Proteomic analysis reveals a role for Bcl2-associated athanogene 3 and major vault protein in resistance to apoptosis in senescent cells by regulating ERK1/2 activation. Mol Cell Proteomics.

[29] Rakha EA, El-Sayed ME, Green AR, Lee AH, Robertson JF, Ellis IO (2007). Prognostic markers in triple-negative breast cancer. Cancer.

[30] Lehmann BD, Bauer JA, Chen X, Sanders ME, Chakravarthy AB, Shyr Y, Pietenpol JA (2011). Identification of human triple-negative breast cancer subtypes and preclinical models for selection of targeted therapies. J Clin Invest.

[31] Kallifatidis G, Munoz D, Singh RK, Salazar N, Hoy JJ, Lokeshwar BL (2016). β-Arrestin-2 Counters CXCR7-Mediated EGFR Transactivation and Proliferation. Mol Cancer Res.

[32] Craig DW, O’Shaughnessy JA, Kiefer JA, Aldrich J, Sinari S, Moses TM, Wong S, Dinh J, Christoforides A, Blum JL, Aitelli CL, Osborne CR, Izatt T (2013). Genome and transcriptome sequencing in prospective metastatic triple-negative breast cancer uncovers therapeutic vulnerabilities. Mol Cancer Ther.

[33] Ammirante M, Rosati A, Arra C, Basile A, Falco A, Festa M, Pascale M, d’Avenia M, Marzullo L, Belisario MA, De Marco M, Barbieri A, Giudice A (2010). IKK{gamma} protein is a target of BAG3 regulatory activity in human tumor growth. Proc Natl Acad Sci USA.

[34] Gentilella A, Khalili K (2011). BAG3 expression in glioblastoma cells promotes accumulation of ubiquitinated clients in an Hsp70-dependent manner. J Biol Chem.

[35] Westhoff MA, Serrels B, Fincham VJ, Frame MC, Carragher NO (2004). SRC-mediated phosphorylation of focal adhesion kinase couples actin and adhesion dynamics to survival signaling. Mol Cell Biol.

[36] Nakai K, Hung MC, Yamaguchi H (2016). A perspective on anti-EGFR therapies targeting triple-negative breast cancer. Am J Cancer Res.

[37] Carey LA, Dees EC, Sawyer L, Gatti L, Moore DT, Collichio F, Ollila DW, Sartor CI, Graham ML, Perou CM (2007). The triple negative paradox: primary tumor chemosensitivity of breast cancer subtypes. Clin Cancer Res.

[38] Brand TM, Iida M, Dunn EF, Luthar N, Kostopoulos KT, Corrigan KL, Wleklinski MJ, Yang D, Wisinski KB, Salgia R, Wheeler DL (2014). Nuclear epidermal growth factor receptor is a functional molecular target in triple-negative breast cancer. Mol Cancer Ther.

[39] Carlsson J, Coleman RG, Setola V, Irwin JJ, Fan H, Schlessinger A, Sali A, Roth BL, Shoichet BK (2011). Ligand discovery from a dopamine D3 receptor homology model and crystal structure. Nat Chem Biol.

[40] Wang AM, Miyata Y, Klinedinst S, Peng HM, Chua JP, Komiyama T, Li X, Morishima Y, Merry DE, Pratt WB, Osawa Y, Collins CA, Gestwicki JE (2013). Activation of Hsp70 reduces neurotoxicity by promoting polyglutamine protein degradation. Nat Chem Biol.

[41] Thomas PD, Campbell MJ, Kejariwal A, Mi H, Karlak B, Daverman R, Diemer K, Muruganujan A, Narechania A (2003). PANTHER: a library of protein families and subfamilies indexed by function. Genome Res.

