# Correction: BAG3 promotes tumour cell proliferation by regulating EGFR signal transduction pathways in triple negative breast cancer

**DOI:** 10.18632/oncotarget.26920

**Published:** 2019-05-03

**Authors:** Sarah Shields, Emer Conroy, Tony O’Grady, Alo McGoldrick, Kate Connor, Mark P. Ward, Zivile Useckaite, Eugene Dempsey, Rebecca Reilly, Yue Fan, Anthony Chubb, David Gomez Matallanas, Elaine W. Kay, Darran O’Connor, Amanda McCann, William M. Gallagher, Judith A. Coppinger

**Affiliations:** ^1^ UCD School of Biomolecular and Biomedical Science, University College Dublin, Dublin 4, Ireland; ^2^ UCD School of Medicine, University College Dublin, Dublin 4, Ireland; ^3^ Systems Biology Ireland, University College, Dublin 4, Ireland; ^4^ Royal College of Surgeons in Ireland, Dublin 2, Ireland; ^5^ Royal College of Surgeons in Ireland, Beaumont Hospital, Dublin 9, Ireland; ^6^ School of Biology and Environmental Science, University College Dublin, Dublin 4, Ireland

**This article has been corrected:** The correct Acknowledgments and Funding information is given below:

**ACKNOWLEDGMENTS AND FUNDING**

This work was supported by the Science Foundation of Ireland, School of Biomolecular and Biomedical Sciences at University College Dublin and the Irish Cancer Society Collaborative Cancer Research Centre BREAST-PREDICT. We would like to acknowledge the National Children’s Research Centre and the Royal College of Surgeons in Ireland for their support. We would also like to thank the Proteomics/Confocal Microscopy Cores, University College Dublin, We would like to thank St Vincent’s University Hospital for their collaboration on the TNBC Patient Cohort (Cohort 3). Finally we would like to acknowledge servier.com for templates used in creating Figure 5E.

Original article: Oncotarget. 2018; 9:15673-15690. https://doi.org/10.18632/oncotarget.24590

