# Supplementary material for: BAG3 promotes tumour cell proliferation by regulating EGFR signal transduction pathways in triple negative breast cancer
Source: Oncotarget. 2018 Feb 28;9(21):15673–90. doi: 10.18632/oncotarget.24590 (PMC5884656; doi:10.18632/oncotarget.24590)
Supplement: Supplementary file 1 [file oncotarget-09-15673-s001.pdf]

BAG3 promotes tumour cell proliferation by regulating EGFR signal transduction pathways in triple negative breast cancer

SUPPLEMENTARY MATERIALS

A

TNBC Subtype	Cell line	Histology	Mutations	Intrinsic Subtype	Basal Subtype
**Basal Like -1**	HCC-1937	DC	BRCA1,TP53, MAPK13,MDC1	HER2	Basal A
	HCC- 1143	IDC	TP53	Basal	Basal A
	HCC-38	DC	CDKN2A, TP53	Unclassified	Basal B
	MDA-MB-468	DC	PTEN, RB1, SMAD4, TP53	Basal	Basal A
**Basal Like-2**	HCC-70	DC	PTEN TP53	Basal	Basal A
**Mesencyhmal**	BT-549	IDC	PTEM, RB1, TP53	Unclassified	Basal B
**Mesencyhmal Stem Like**	MDA-MB-436	IDC	BRCA1, TP53	Unclassified	Basal B
	MDA-MB-231	IDC	BRAF, CDKN2A, KRAS, NF2, TP53, PDGFRA	Unclassified	Basal B

B

	**Low Bag3 0,1+)**	**High Bag3 (2+, 3+)**	**Significance**
**No of TNBC Patients**	41	37	p values-Fisher exact
**Patient Age**	32-86	25-78	
**Nodal Status**			
Negative	21 (51.2%)	23 (62.2%)	
Positive	16 (39%)	14 (37.8%)	
Unclassified	4 (9.8%)	0(0%)	0.81
**Accompanying DCIS**			
IDC alone	8 (19.5%)	14 (37.8%)	
IDC +DCIS	29 (70.7%)	22 (59.5%)	0.13
Unclassified	4(9.8%)	1 (2.7%)	
**Chemotherapy**			
No	17 (41.5%)	7 (19.0%)	
Yes (Taxane or other)	24 (58.5%)	30 (81.0%)	0.04
**Radiotherapy**			
No	9 (22.0%)	5 (13.5%)	
Yes	29 (70.7%)	32 (86.5%)	
Unclassified	3 (7.3%)	0 (0%)	0.37

Supplementary Figure 1: (**A**) Assignment of TNBC cell lines to TNBC subtypes, mutations and histologies. DC represents, ductal carcinoma and IDC represents invasive ductal carcinoma. Table adapted from Lehmann *et al* (31). (**B**) The relationship between BAG3 protein expression and clinical variables in TNBC Cohort 3. *P* values were generated using Fishers exact test, $p < 0.05$ denotes significance. DCIS represents ductal carcinoma *in situ*.

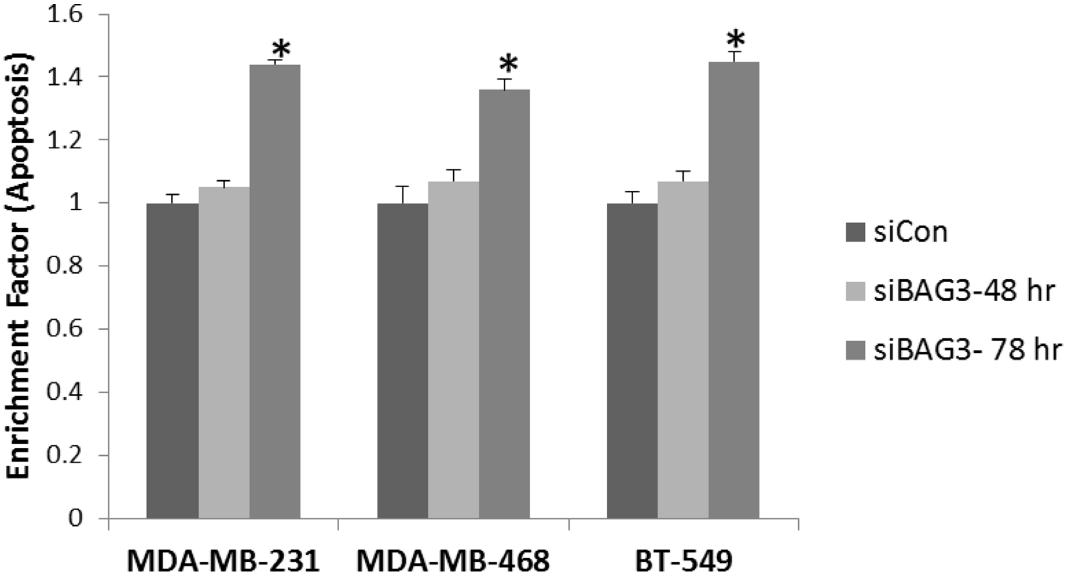

Supplementary Figure 2: A quantitative graph of apoptosis (mononucleasome enrichment) in MDA-MB-468, MDA-MB-231 and BT-549 cell lines treated with siBAG3 and siControl for 48 and 72 hrs.

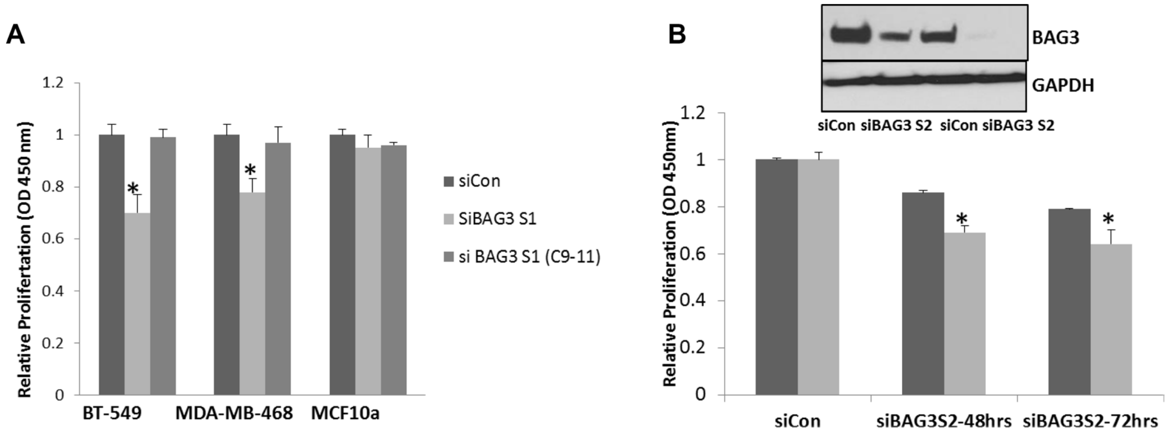

Supplementary Figure 3: (**A**) To further ensure this siRNA was specific, a rescue experiment was performed with a (C9-C11) version of the BAG3 (S1) siRNA sequence in TNBC (MDA-MB-468, BT-549) and controls cells (MCF10a). The effects on proliferation between the selected siRNA targeting BAG3 (S1) and its mutant (C9-C11) were compared. (**B**) Additionally MDA-MB-468 and BT-549 cells were transfected with a second siRNA sequence targeting BAG3 (S2). Reduction of BAG3 was visualised by immunoblotting and effects on proliferation with a second siRNA measured as before. The histograms represent mean BrdU (±SD) incorporation relative to the control.

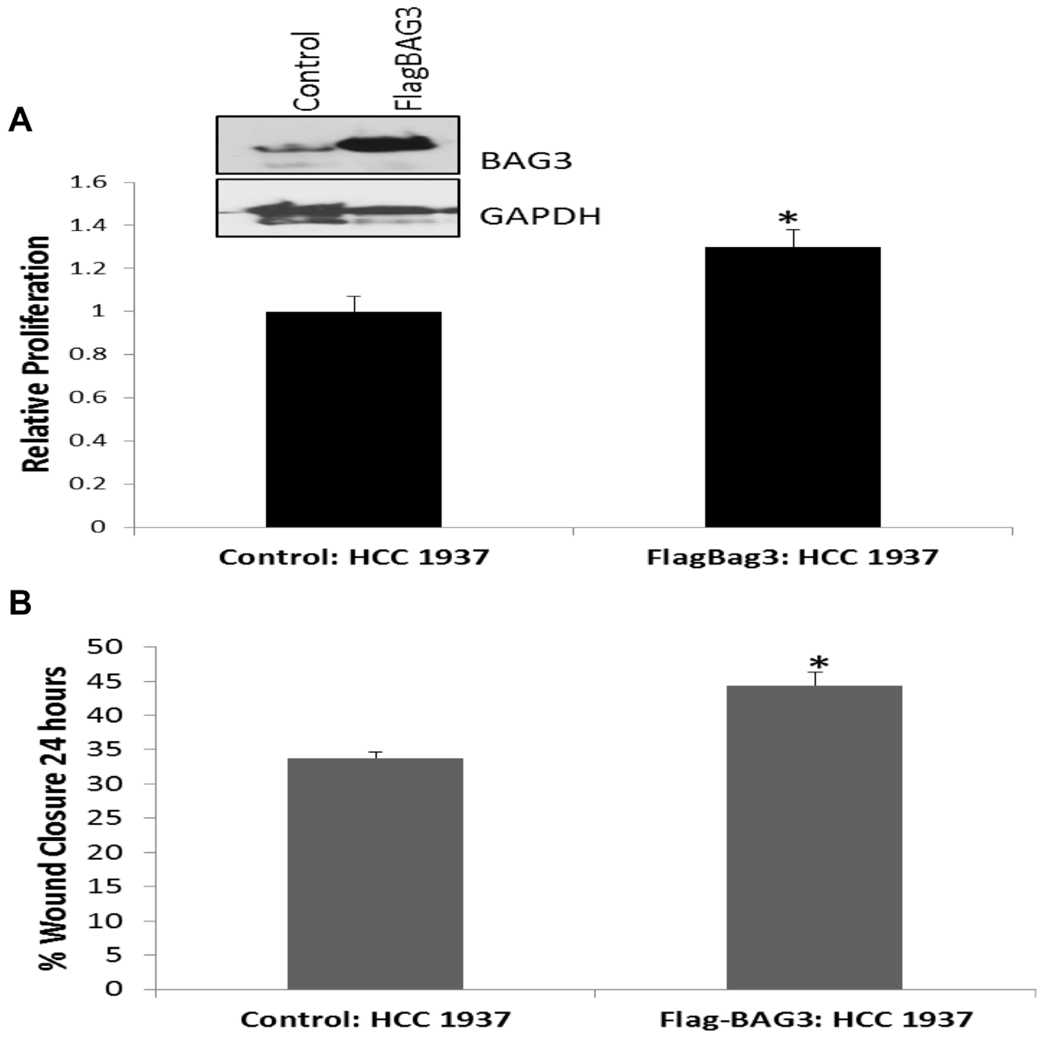

Supplementary Figure 4: (**A**) The HCC1937 cell line was transfected with FlagBAG3 or vector control and immunoblotting was performed for BAG3 and GAPDH. Proliferation was measured in HCC1937 cells treated with FlagBAG3 and control. The histograms represent mean (± SD) Brdu incorporation relative to the control (n = 3). (**B**) A quantitative graph of percentage wound closure from HCC1937 cells after treatment with FlagBAG3 or control. The histograms represent average wound closure (± SD). An asterisk represents $p < 0.05$.

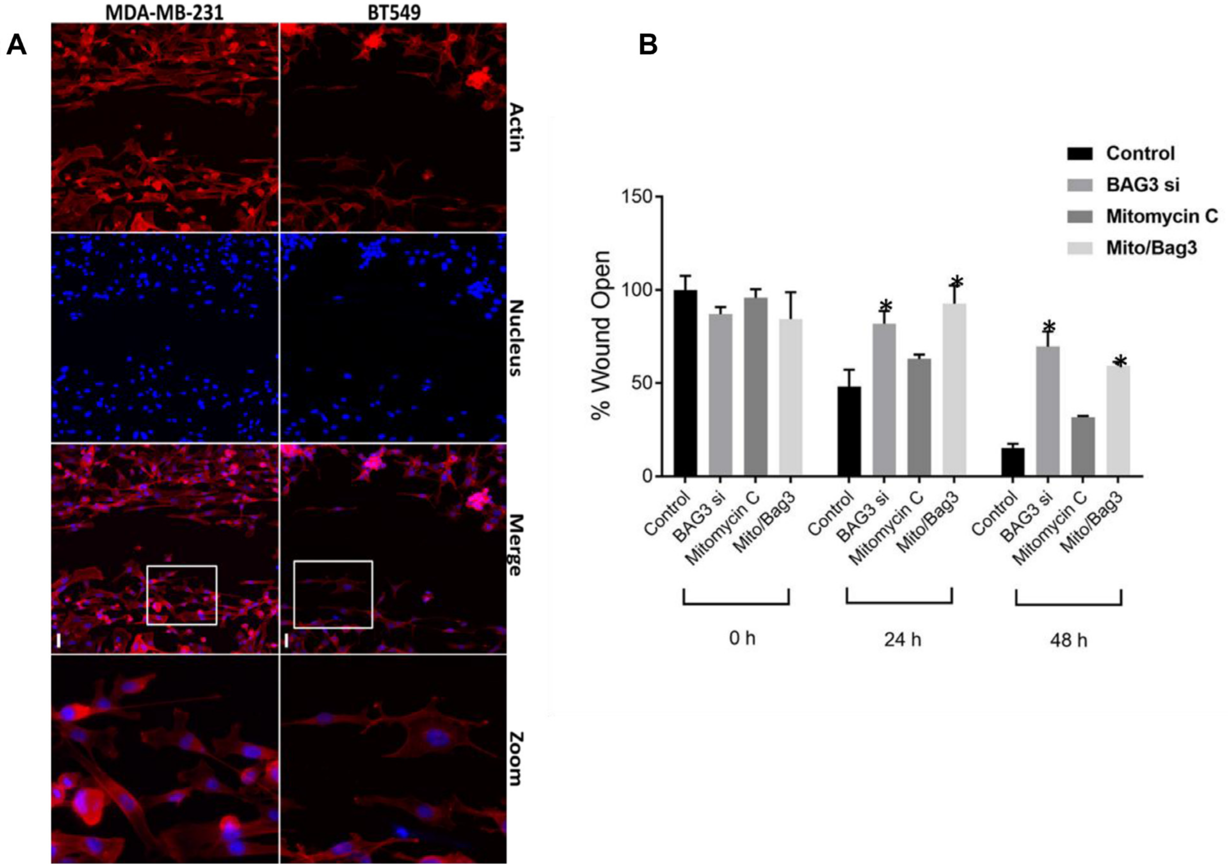

Supplementary Figure 5: (**A**) Wounds were created for 24 hrs in BT-549 and MDA-MB-231 cells and the migrating cells were demonstrated by immunofluorescence. Actin was stained using AlexaFluor568 labelled phalloidin and Hoechst 33342 was used to visualise the nuclei. Scale bar = 50 µm, white box represents zoom area. Arrow heads indicate regions of actin enrichment in lamellipodia indicative of migrating cells. (**B**) MDA-MB-468 cells were treated with siBAG3 and siControl in the presence and absence of 5 uM Mitomycin C. A scratch wound assay was performed as before for 24 and 48 hrs. A quantitative graph of percentage wound opening under the different conditions is displayed. The histograms represent % wound opening ± SD. An asterisk represents $p < 0.05$.

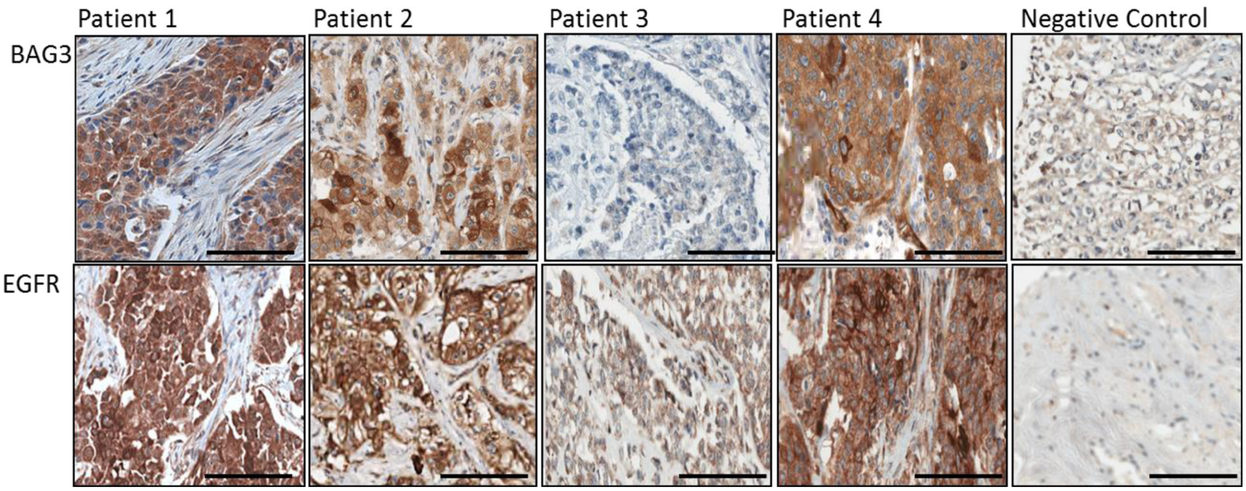

Supplementary Figure 6: Representative scanned images of tumour sections from 4 TNBC patients with low or high BAG3 and EGFR expression as determined by immunohistochemistry (*n* = 10). Scale bar is 100 uM. Magnification is 20×.

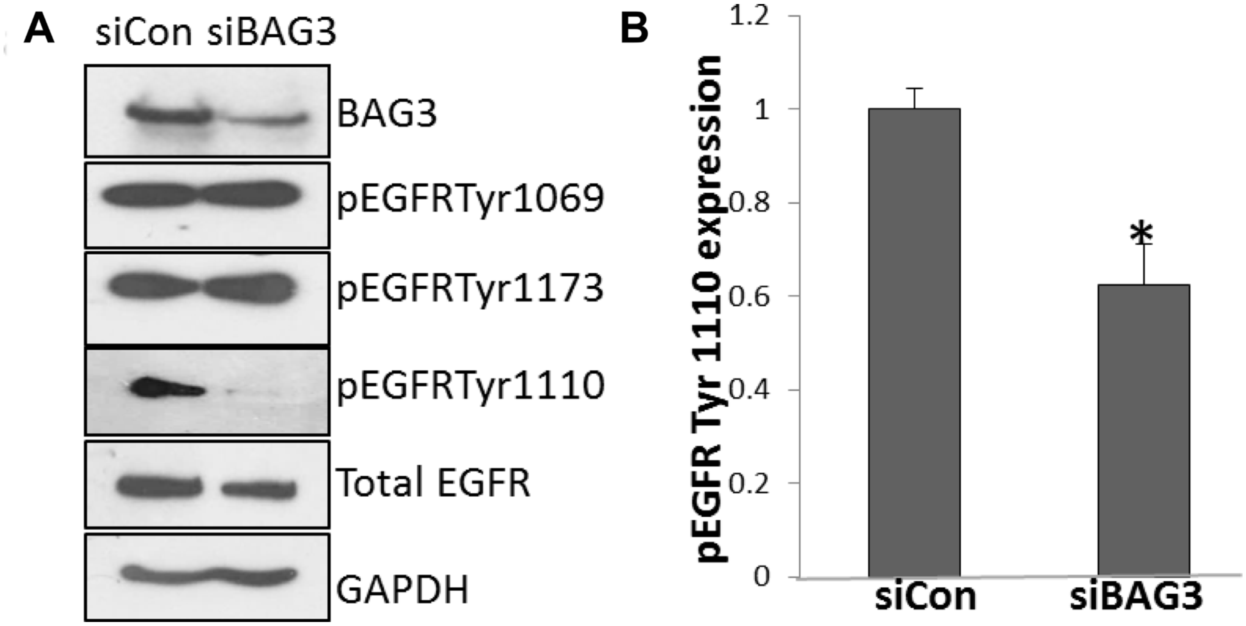

Supplementary Figure 7: MDA-MB-468 cells treated with siControl and siBAG3. (**A**) The protein expression of pEGFRTyr1069, pEGFRTyr1173, pEGFRTyr1110, EGFR and BAG3 was analysed by immunoblotting. (**B**) The histograms represent average protein expression of pEGFRTyr1110 ± SD (*n* = 3) relative to the control. For all experiments an asterisk represents $p < 0.05$.

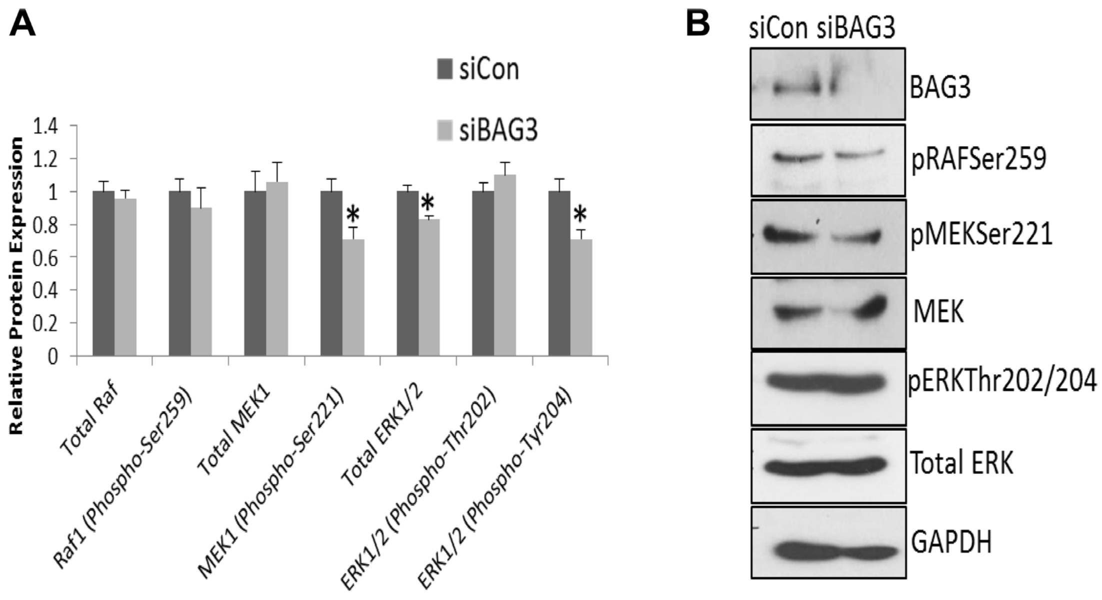

Supplementary Figure 8: (**A**) The quantitative histograms of the Raf/MEK/ERK pathways represent the average of 6 replicate probes normalized to Actin and the error bars represent the standard deviation. (**B**) The protein expression of BAG3, pRafSer289/296/301, pMEKSer221, MEK, pERKThr202/204, ERK and GAPDH were confirmed by immunoblotting.

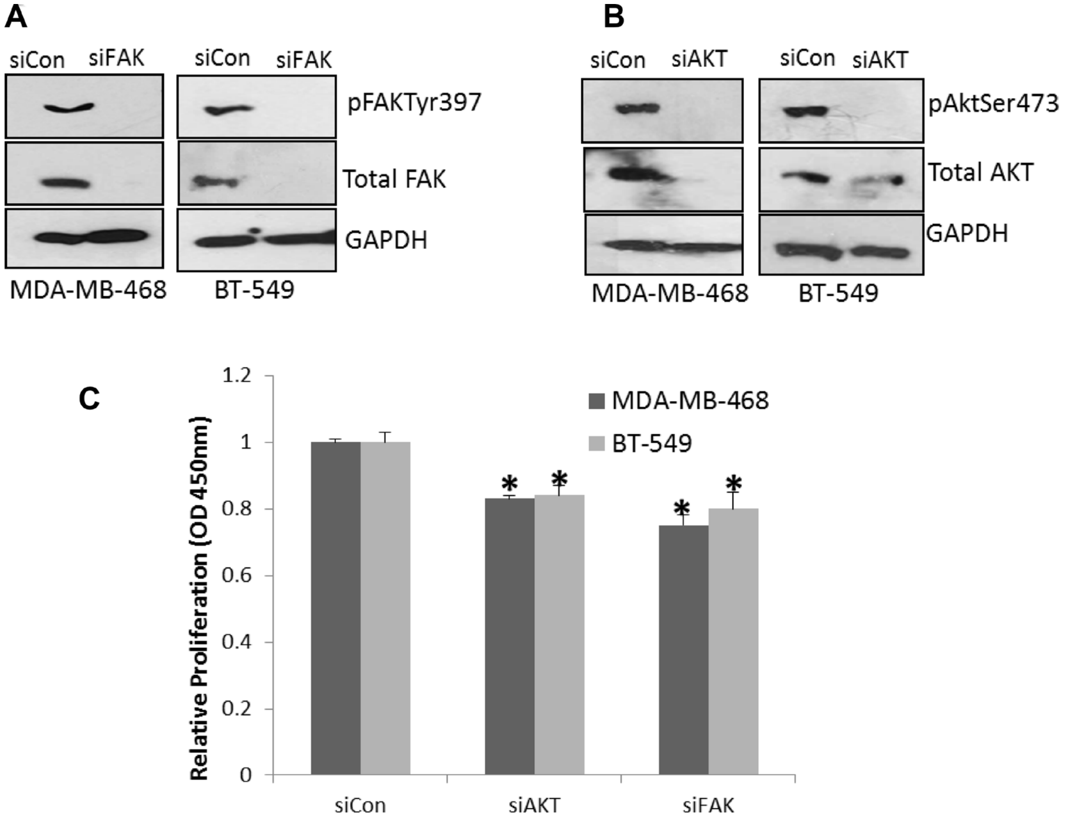

Supplementary Figure 9: (**A**) BT-549 and MDA-MB-468 cell lines were transfected with siAKT and siFAK1 for 48 hours. Reduction of FAK1 protein expression after gene silencing was confirmed by immunoblotting. (**B**) Reduction of AKT1 protein expression after gene silencing was confirmed by immunoblotting. (**C**) A quantitative graph of proliferation in MDA-MB-468, and BT-549 cell lines after treating with siFAK1 and siAKT1.The histograms represent mean (±SD) Brdu incorporation relative to the control ±SD ($n = 3$). An asterisk represents $p < 0.05$.

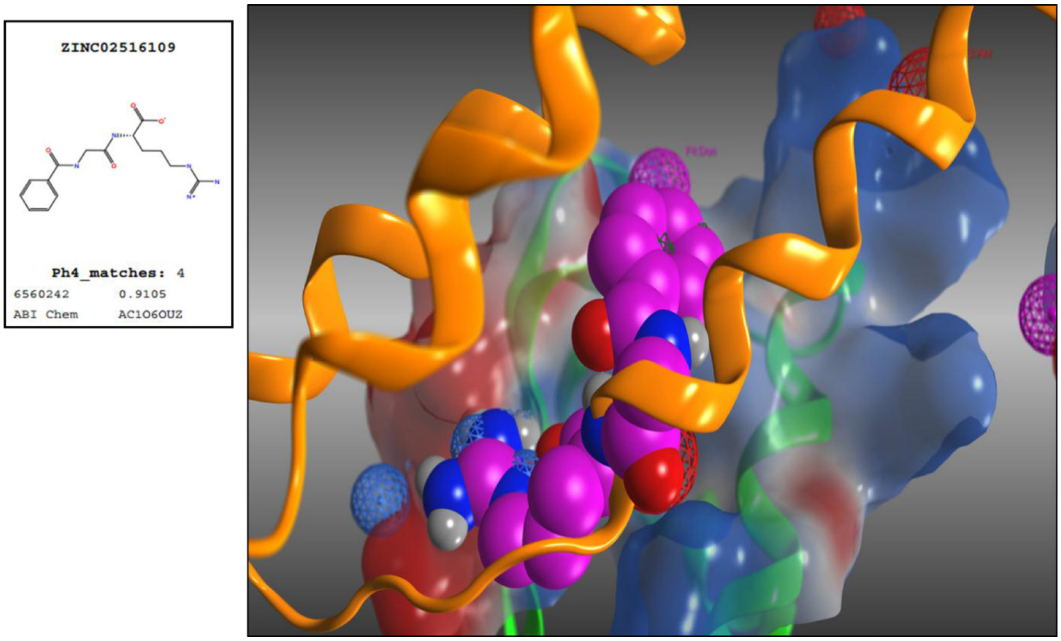

Supplementary Figure 10: A hit from the virtual high throughput screen (ZINC20516109) is shown with pink carbons, blue nitrogens, red oxygens and white hydrogens. The pharmacophore spheres used in the selection are shown with wire mesh, where cations are light blue, anions are red and hydrophobic regions are green. The electrostatic surface human Hsp70 (green ribbon) that binds to Bag3 (brown ribbon) is shown with positive regions blue and negative regions red.

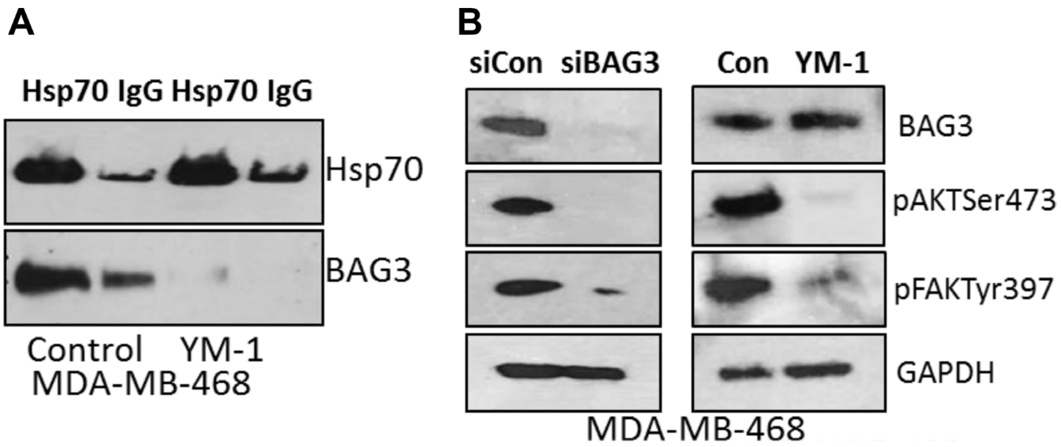

Supplementary Figure 11: (**A**) The MDA-MB-468 cell line was treated with YM-1 (5 uM) and co-immunoprecipitation of Hsp70 and BAG3 was performed. The expression of BAG3 and Hsp70 as determined by immunoblotting after co-immunoprecipitation is displayed. Co-immunoprecipitation of Hsp70 and BAG3 from MDA-MB-468 cell lysates showed that YM-1 disrupts formation of the Hsp70-Bag3 complex. (**B**) MDA-MB-468 cells were treated with siBAG3, siControl, DMSO or 5 uM YM-1 for 48 hours. The protein expression of BAG3, pAKTSer473, pFAKTyr397, and GAPDH was determined by immunoblotting.

Supplementary Table 1: BAG3 interacting proteins identified by mass spectrometry in TNBC cell lines; BT-549 and MDA-MB-468. The proteins names, mass, peptide count and sequence coverage are listed. See Supplementary_Table_1

Supplementary Table 2: The Cy3 fluorescent intensity values of 214 antibodies on the EGF phosphoarray from MDA-MB-468 cells treated with siBAG3 and siControl are listed. See Supplementary_Table_2
